# Congo Basin Carbon Cycle Responses to Global Change

**DOI:** 10.1111/gcb.70688

**Published:** 2026-01-20

**Authors:** Sarah Worden, Rong Fu, A. Anthony Bloom, Marijn Bauters, Hans Verbeeck, Temilola Fatoyinbo, Wannes Hubau, Lydie‐Stella Koutika, Steve Kwatcho Kengdo, Sybryn L. Maes, Vincent Medjibe, Nicholas J. Russo, Sassan Saatchi, Le Bienfaiteur Sagang, Thomas B. Smith, Denis J. Sonwa, Pascal Boeckx, Elsa M. Ordway

**Affiliations:** ^1^ Jet Propulsion Laboratory California Institute of Technology Pasadena California USA; ^2^ Atmospheric and Oceanic Sciences Department University of California, Los Angeles Los Angeles California USA; ^3^ Q‐ForestLab, Department of Environment Ghent University Ghent Belgium; ^4^ Massachusetts Institute of Technology Program in Media Arts and Sciences Cambridge Massachusetts USA; ^5^ Laboratory of Wood Technology, Department of Environment Ghent University Ghent Belgium; ^6^ Service of Wood Biology Royal Museum for Central Africa Tervuren Belgium; ^7^ Research Center (CRDPI/SCES) Pointe‐Noire Republic of the Congo; ^8^ Earth and Environmental Sciences Area Lawrence Berkeley National Laboratory Berkeley California USA; ^9^ Forest Ecology and Management Group (FORECOMAN), Department of Earth and Environmental Sciences KU Leuven Leuven Belgium; ^10^ Independent Researcher Libreville Gabon; ^11^ Department of Organismic and Evolutionary Biology Harvard University Cambridge Massachusetts USA; ^12^ Institute of the Environment and Sustainability University of California, Los Angeles Los Angeles California USA; ^13^ Department of Ecology and Evolutionary Biology University of California, Los Angeles Los Angeles California USA; ^14^ World Resources Institute (WRI) Kinshasa Democratic Republic of the Congo; ^15^ Isotope Bioscience Laboratory Ghent University Ghent Belgium

**Keywords:** animal–ecosystem interactions, carbon cycle, climate change, CO_2_ fertilization, land cover and land use change, legacy effects, nutrient availability

## Abstract

The Congo Basin and its contiguous forests harbor globally significant carbon stocks, estimated at 65 gigatons of C (GtC) above and belowground. Despite rising temperatures and intensifying droughts, they have remained a carbon sink, albeit weak: 0.26–0.50 GtC yr.^−1^ carbon uptake since 1980. However, these forests' carbon stocks and fluxes, including gross primary productivity, respiration, net primary productivity, and riverine carbon transport, remain poorly quantified. This limits understanding of the region's role in the global carbon cycle, its vulnerability to environmental change, and its potential as a long‐term carbon sink. We review and quantify Congo Basin and contiguous forest carbon stocks and fluxes and synthesize the current knowledge on how key global change drivers shape the region's carbon cycle. We find limited responses to long‐term precipitation variability, but declining stocks and fluxes in response to long‐term and increasing temperature and drought frequency. Land cover and land use changes, largely driven by small‐scale agriculture, logging, and agro‐industrial expansion, reduce carbon stocks, ecosystem structure and functioning, and animal‐mediated ecosystem services. In contrast, large‐scale savanna biomass burning delivers phosphorus and nitrogen to Congo Basin forests via cross‐equatorial winds, providing additional nutrients and supporting carbon sequestration. In situ studies suggest that CO_2_ fertilization has increased intrinsic water‐use efficiency, although its effects may be modulated by climate change, and its impacts on biomass accumulation remain uncertain. Legacy effects from historical land use and climate change likely shaped present‐day vegetation structure, yet their relative influence is unclear. High‐resolution carbon monitoring, improved remote sensing, and strengthened in situ measurement networks are needed to quantify the impacts of these key drivers and their interactions on the Central African carbon cycle. This is needed to inform conservation strategies and advance understanding of the region's future as a carbon sink under global change pressures.

## Graphical abstract

Understanding the resilience of the Congo Basin carbon cycle in the face of current and future pressures requires a detailed understanding of its response to past and present drivers of change. Building on a pantropical framework of drivers and possible regional mechanisms for resilience, this review provides the first comprehensive assessment of Congo Basin carbon cycle responses to change, focusing mainly on the late 20th century to present. We first quantify and describe three components of the Congo Basin carbon cycle: (1) above‐ and belowground carbon stocks, (2) GPP, net primary productivity (NPP) and respiration (total, autotrophic, or heterotrophic), and (3) lateral carbon fluxes. We then synthesize current knowledge on the response of these components to the four drivers of change: climate change, land cover and land use change (LCLUC), carbon dioxide (CO_2_) fertilization, and legacy effects, as well as their interactions. We conclude by identifying priorities for future research.

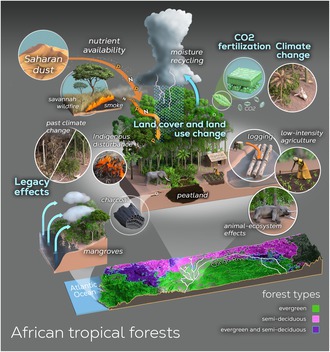

## RÉSUMÉ

Le bassin du Congo et ses forêts adjacentes abritent des stocks de carbone d’importance mondiale, estimés à 65 gigatonnes de carbone (GtC) en surface et en sous‐sol. Malgré la hausse des températures et l’intensification des sécheresses, la région reste un puits de carbone, bien que faible: une absorption de 0,26 à 0,50 GtC par an depuis 1980. Toutefois, les stocks et flux de carbone de ces forêts — incluant la productivité primaire brute, la respiration, la productivité primaire nette et le transport fluvial de carbone — restent mal quantifiés. Cela limite la compréhension du rôle de la région dans le cycle mondial du carbone, de sa vulnérabilité face aux changements environnementaux, et de son potentiel en tant que puits de carbone à long terme. Nous passons en revue et quantifions les stocks et flux de carbone du bassin du Congo et des forêts adjacentes, et synthétisons les connaissances actuelles sur l’influence des principaux facteurs du changement global sur le cycle du carbone régional. Nous constatons une réponse limitée à la variabilité à long terme des précipitations, mais des diminutions des stocks et flux en lien avec la hausse des températures et la fréquence accrue des sécheresses. Les changements d’occupation et d’utilisation des sols, principalement dus à l’agriculture à petite échelle, à l’exploitation forestière et à l’expansion agro‐industrielle, réduisent les stocks de carbone, la structure et le fonctionnement des écosystèmes, ainsi que les services écosystémiques assurés par la faune. La combustion de biomasse des savanes à grande échelle apporte du phosphore et de l’azote aux forêts du bassin du Congo via les vents transéquatoriaux, fournissant des nutriments supplémentaires à la région et soutenant le stockage de carbone. Des études in situ suggèrent que la fertilisation par le CO_2_ a accru l’efficacité intrinsèque de l’utilisation de l’eau, bien que ses effets puissent être modulés par le changement climatique, et son impact sur l’accumulation de biomasse reste incertain. Les effets hérités de l’utilisation passée des terres et du changement climatique ont probablement façonné la structure actuelle de la végétation, mais leur influence relative demeure floue. Une surveillance du carbone à haute résolution, une télédétection améliorée et un renforcement des réseaux de mesures in situ sont nécessaires pour quantifier les effets de ces facteurs clés et de leurs interactions sur le cycle du carbone en Afrique centrale. Cela est indispensable pour guider les stratégies de conservation et améliorer notre compréhension de l’avenir de la région en tant que puits de carbone face aux pressions du changement global.

## 1 Introduction

The Congo Basin and its contiguous tropical forests hold one of the world's largest and least understood carbon reserves, including ~36 ± 0.26 Gigatons of C (GtC) in forests (~9.4% of global live biomass) and ~29 ± 3 GtC in peatlands (~1.6% of global soil carbon) (Crezee et al. 2022; Saatchi et al. 2011; Verhegghen et al. 2012; Xu et al. 2021). However, climate and land‐use changes are rapidly altering the region's forest structure, function, and biodiversity. Recent decades have seen rising drought vulnerability (Tao et al. 2022), tree mortality (Hubau et al. 2020), and fire activity (Wimberly et al. 2024), alongside growing demands from foreign resource interests and population increases (Gerland et al. 2014; Ezeh et al. 2020; Fuller et al. 2019; Sovacool 2019; Ordway, Naylor, et al. 2019). These pressures are accelerating deforestation and degradation due to small‐scale agriculture, agro‐industry, and logging (Tyukavina et al. 2018; Sagang et al. 2024; Shapiro et al. 2023; Feintrenie 2014; Biswas et al. 2025).

Despite these pressures, the region's moist tropical forests have likely remained a weak aboveground carbon sink (net uptake minus emissions between 2000 and 2019: 0.02 GtC yr.^−1^; Xu et al. 2021; between 2010 and 2019: 0.004 GtC yr.^−1^; Zhao, Ciais, et al. 2024). Remotely sensed estimates measure 0.26 GtC yr.^−1^ of carbon uptake (2000–2019) (Xu et al. 2021), while ground‐based estimates measure 0.28–0.50 GtC yr.^−1^ (1980–2020) (Hubau et al. 2020; Lewis et al. 2009). Aboveground biomass (AGB) has increased since the 1980s (Lewis et al. 2009; Xu et al. 2021), in contrast to AGB declines in the Amazon and Indonesian rainforests (Xu et al. 2021). The Congo Basin is projected to remain a net carbon sink for decades, while the Amazon may cease being a net carbon sink by the 2030's (Hubau et al. 2020). However, focusing solely on aboveground carbon dynamics risks masking critical belowground carbon (potentially larger than aboveground carbon; Doetterl et al. 2016) vulnerabilities. Soil carbon losses, nutrient depletion and export, shifting fire regimes, and peatland drying are poorly monitored, and belowground carbon may respond differently to these pressures compared to aboveground carbon (Jones et al. 2019).

How the Congo Basin's carbon cycle will respond to accelerating 21st‐century pressures remains unknown. Assessing this is challenging due to limited research investment, infrastructure, and sparse in situ data collection compared to the rest of the tropics and the world, leaving Congo Basin forests among the least understood regions (White et al. 2021; Alsdorf et al. 2016; Tshimanga et al. 2022). Earth System models predicting intensified biogeochemical and hydrological cycles in the region lack sufficient in situ meteorological and surface observations to accurately represent the feedbacks between these cycles (e.g., Green et al. 2019; Neelin et al. 2006; Bonan 2008; Bonan and Doney 2018; Worden, Saatchi, et al. 2021).

**FIGURE 1:**
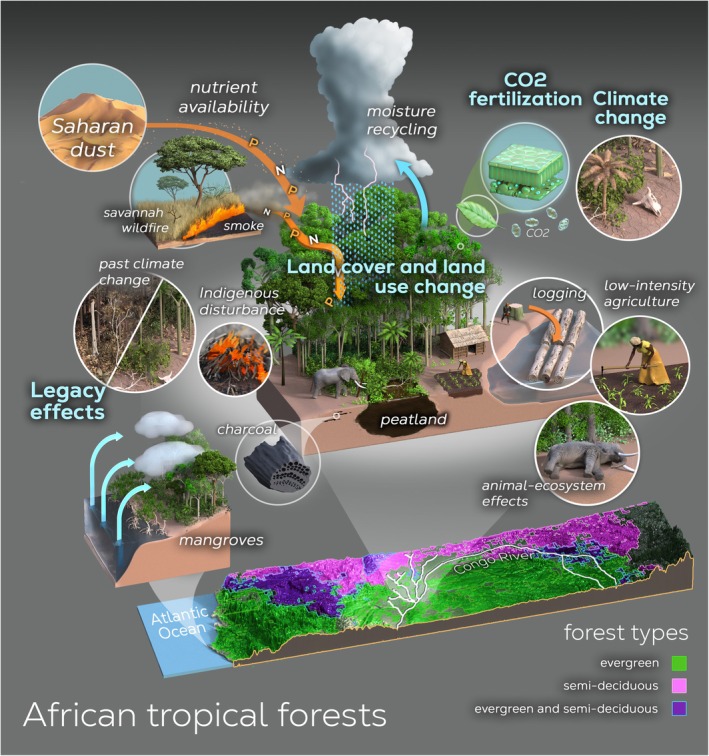
Illustration of four drivers that have changed Congo Basin carbon stocks and fluxes: (1) climate change (precipitation, temperature, drought, and ENSO), (2) land cover and land use change (deforestation and degradation, animal‐ecosystem impacts, remote impacts of large scale biomass burning), (3) $CO_{2}$ fertilization (impacts on water use efficiency), and (4) legacy effects (past climate and past disturbances on the scale of 5000 years to present).

Significant uncertainties persist around the Congo Basin carbon cycle trajectory under climate and human pressures (e.g., Burnett et al. 2020; Ciais et al. 2011; Pan et al. 2024). Key drivers include climate change, land cover and land use change (LCLUC), carbon dioxide ($CO_{2}$) fertilization, and legacy effects (Figure 1). Carbon stocks and fluxes respond to shifts in temperature, precipitation, and growing seasons (e.g., McDowell et al. 2018; Tharammal et al. 2019). LCLUC, such as deforestation, degradation, and biomass burning, impact biogeochemical processes and increase vegetation regime shift risks (e.g., Tharammal et al. 2019). Animal–vegetation interactions further shape vegetation structure, nutrient cycling, and ecosystem function (e.g., Malhi et al. 2022; Russo et al. 2023). Rising atmospheric $CO_{2}$ can enhance gross primary productivity (GPP) through increased water use efficiency (WUE) (e.g., Eamus 1991; Keenan et al. 2013; Ueyama et al. 2020; but see Zhan et al. 2025). Legacy effects, defined here as ecosystem responses to disturbances within the past 5000 years, can influence current carbon dynamics (e.g., Kannenberg et al. 2020; Krause et al. 2020). These drivers can interact synergistically or antagonistically, yielding complex net effects on the Congo Basin carbon cycle.

Understanding the resilience of the Congo Basin carbon cycle in the face of current and future pressures requires a detailed understanding of its response to past and present drivers of change (Malhi et al. 2014). Building on a pantropical framework of drivers (Malhi et al. 2014) and possible regional mechanisms for resilience (Bennett et al. 2021; Hubau et al. 2020), this review provides the first comprehensive assessment of Congo Basin carbon cycle responses to change, focusing mainly on the late 20th century to present. We first quantify and describe three components of the Congo Basin carbon cycle: (1) above‐ and belowground carbon stocks, (2) GPP, net primary productivity (NPP) and respiration (total, autotrophic, or heterotrophic), and (3) lateral carbon fluxes (Table 1). We then synthesize current knowledge on the response of these components to the four drivers of change. We conclude by identifying priorities for future research.

**TABLE 1:** Definitions and summarization of uncertainties associated with the three carbon cycle components reviewed, including stocks (blue), fluxes (green; gross primary productivity (GPP), respiration, and net primary productivity (NPP)), and lateral carbon flux (orange; dissolved organic carbon (DOC), dissolved inorganic carbon (DIC), particulate organic carbon (POC), and $CO_{2}$ evasion).

	Name	Definition	Units used in this paper	Attributed uncertainties
Stock	Aboveground/belowground biomass (AGB/BGB)	The total amount of aboveground/belowground living organic matter expressed as mass	Pg, ton (t)	**AGB/AGC**: *Medium evidence*: *G*reater availability of direct measurements compared to fluxes *Medium agreement*: Greater spatio‐temporal agreement between datasets compared to fluxes and belowground stocks **BGB/BGC**: *Medium evidence*: Greater availability of direct measurements compared to fluxes *Low agreement*: Reliance on modeled upscaling for global estimates leads to large dataset divergence References: Araza et al. (2023); El Masri and Xiao (2025)
	AGB/BGB density	AGB/BGB expressed as mass per unit area	Pg ha^−1^, t ha^−1^	
	Aboveground/belowground carbon (AGC/BGC)	The fraction of AGB/BGB that is composed of carbon expressed as mass	PgC, tC, Megaton (Mt) C	
	AGC/BGC density	AGC/BGC expressed as mass per unit area	PgC ha^−1^, tC ha^−1^, MtC ha^−1^	
	Soil organic carbon (SOC)	The amount of carbon stored in nonliving organic matter, expressed as mass	tC	*Medium evidence*: Greater availability of direct measurements compared to fluxes *Low agreement*: Reliance on modeled upscaling for global estimates leads to large dataset divergence References: Wang et al. (2023); Ziqi et al. (2024)
Flux	Gross primary productivity (GPP)	The sum of gross C fixation by autotrophic C‐fixing tissues per unit ground or water area and time *Into the system*	MtC ha^−1^ day^−1^	*Limited evidence*: greater availability of direct measurements compared to fluxes *Low agreement*: Reliance on modeled upscaling for global estimates leads to large dataset divergence References: Hashimoto et al. (2023); Qiu et al. (2025); Stell et al. (2021); Bai et al. (2023); Yang et al. (2021); Zhang and Ye (2021, 2022)
	Autotrophic respiration (AR)	The sum of respiration by all living parts of primary producers per unit ground or water area and time *Out of the system*	MtC ha^−1^ day^−1^	
	Heterotrophic respiration (HR)	The respiration rate of heterotrophic organisms (animals and micros) summed per unit ground or water area and time *Out of the system*	MtC ha^−1^ day^−1^	
	Ecosystem respiration (ER)	The respiration of all organisms summed per unit ground or water area and time *Out of the system*	MtC ha^−1^ day^−1^	
	Net primary production (NPP)	GPP minus autotrophic respiration *In or out of the system*	MtC ha^−1^ day^−1^	
	Dissolved organic carbon (DOC)	Organic carbon that can pass through a filter, usually 0.45 μm. *Laterally through the system*	Mt C yr.^−1^	*Limited evidence*: Limited direct measurements, limited large‐scale modeling *Low agreement*: High uncertainties of measurements References: Raymond et al. (2013); Tian et al. (2023)
	Dissolved inorganic carbon (DIC)	Inorganic carbon that can pass through a filter *Laterally through the system*	MtC yr.^−1^	
	Particulate organic carbon (POC)	Organic carbon that can be collected on a filter *Laterally through the system*	MtC yr.^−1^	
	CO_2_ evasion	CO_2_ dissolved in water that is released to the atmosphere *Out of the system*	MtC yr.^−1^	

*Note:* Most definitions are referenced from Chapin et al. (2006), Brown (1997), and Kolka et al. (2008).

## 2 Study Area and Terminology

**FIGURE 2:**
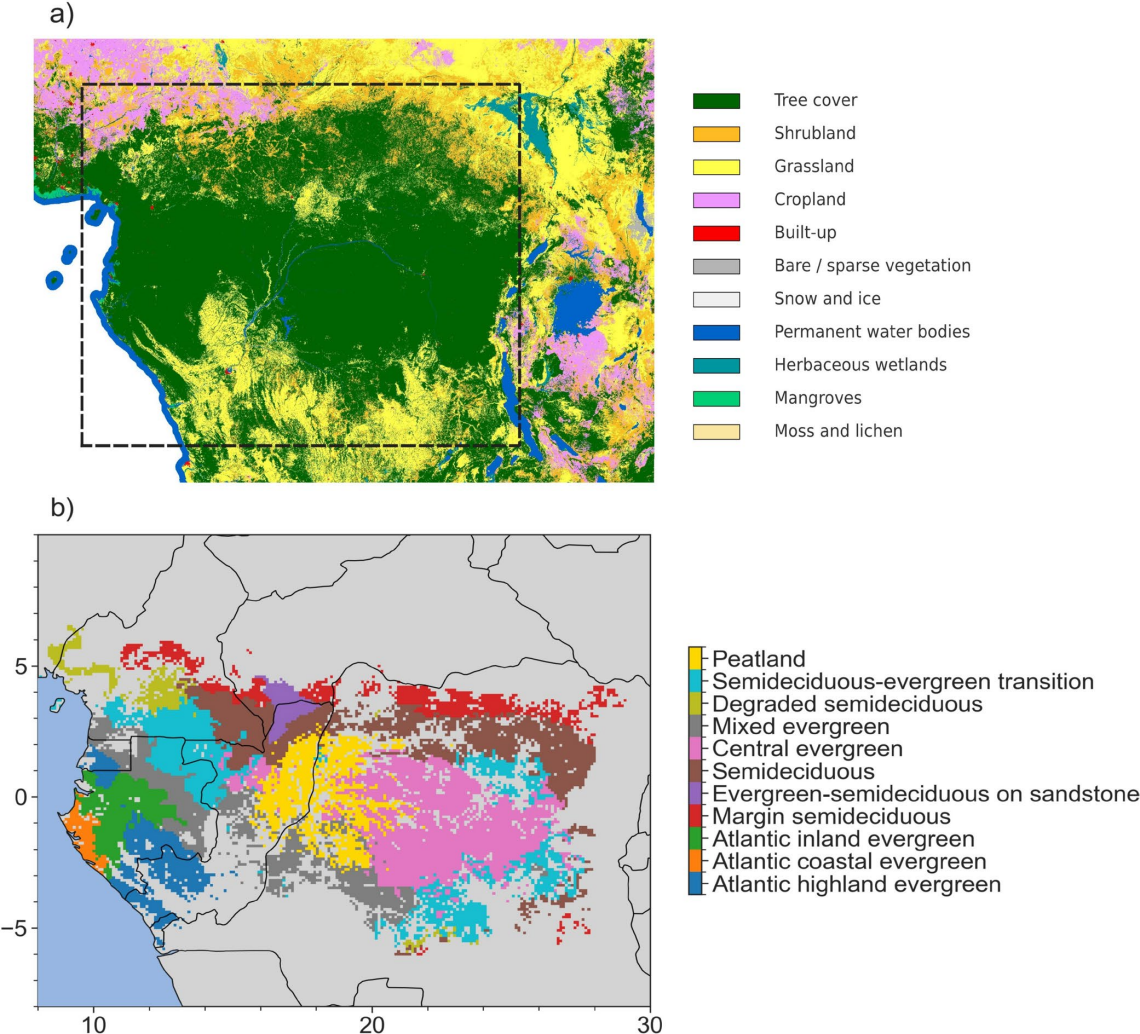
(a) African vegetation types, taken from the ESA WorldCover 10 m 2021 v200 map (Zanaga et al. 2022). Dashed box represents the area of study and (b) Congo Basin forest floristic groups. Data from Réjou‐Méchain et al. (2021a). Map lines delineate study areas and do not necessarily depict accepted national boundaries.

We define the Congo Basin and its contiguous tropical forests as all terrestrial and aquatic ecosystems between 10° N–8° S and 8° E–30° E, spanning parts of Cameroon, Gabon, Equatorial Guinea, Central African Republic (CAR), Republic of Congo (RoC), and the Democratic Republic of the Congo (DRC). This includes savannas and grasslands interspersed with tropical forests and ecotones, and the Congo River, which transports largely forest‐sourced carbon (Drake et al. 2023). Referencing the Congo Basin beyond just the hydrological basin is consistent with terminology used by major science initiatives, including the Science Panel for the Congo Basin and the Congo Basin Science Initiative. This review primarily focuses on forests owing to their disproportionate impact on the region's carbon cycle; however, we also examine shrubland, grassland, cropland, permanent water bodies, and mangroves where important interactions, dynamics, and knowledge gaps exist (Figure 2a; Verhegghen et al. 2012; Réjou‐Méchain et al. 2021b). The region's tropical forest, also referred to as Guineo‐Congolian forest, is typically divided into distinct evergreen and semi‐deciduous floristic groups (Figure 2b), with tree community composition primarily driven by climate and human‐induced disturbance intensity (Réjou‐Méchain et al. 2021b; Fayolle et al. 2014; Ouédraogo et al. 2016; Viennois et al. 2013). We report on vegetation components that are related to, or can affect, carbon cycling, including tree growth, species composition, species biodiversity, and canopy structure. Vegetation types storing significant carbon include peatland forests in the Cuvette Centrale (Alsdorf et al. 2016; Dargie et al. 2019; Crezee et al. 2022), mangroves along the coastline (John and Lawson 1990; Naidoo 2023), and forests with monodominant stands of *Gilbertiodendron dewevrei* (Kearsley et al. 2017; Hall et al. 2020; Heimpel et al. 2024; Glick et al. 2023).

Congo Basin carbon cycle research includes ground‐based field studies, and remote sensing and modeling analyses. Research employing any of these methods, ranging from the site to regional scale, was reviewed. In situ measurements of carbon stocks are primarily from networks like the African Tropical Rainforest Observation Network (AfriTRON, https://afritron.org/), the Central African Plot Network (https://central‐african‐plot‐network.netlify.app/map/), and the Global Ecosystems Monitoring (GEM) network (http://gem.tropicalforests.ox.ac.uk/). Plots in these networks span Gabon, Equatorial Guinea, Cameroon, RoC, DRC, Uganda, and Rwanda (e.g., Lewis et al. 2013). Most plots are concentrated within the western Congo Basin (Figure S1). Currently, in situ estimates of carbon fluxes are extremely limited, with one flux tower established in 2021 at the Yangambi research center in the DRC (Sibret et al. 2022). Additionally, no single repository or long‐term monitoring effort detailing carbon fluxes transported by the Congo River exists. For this reason, studies examining carbon fluxes in the region are limited. The lack of mention of these fluxes in the subsequent sections indicates an absence of research associating changes in fluxes with the corresponding driver.

**FIGURE 3:**
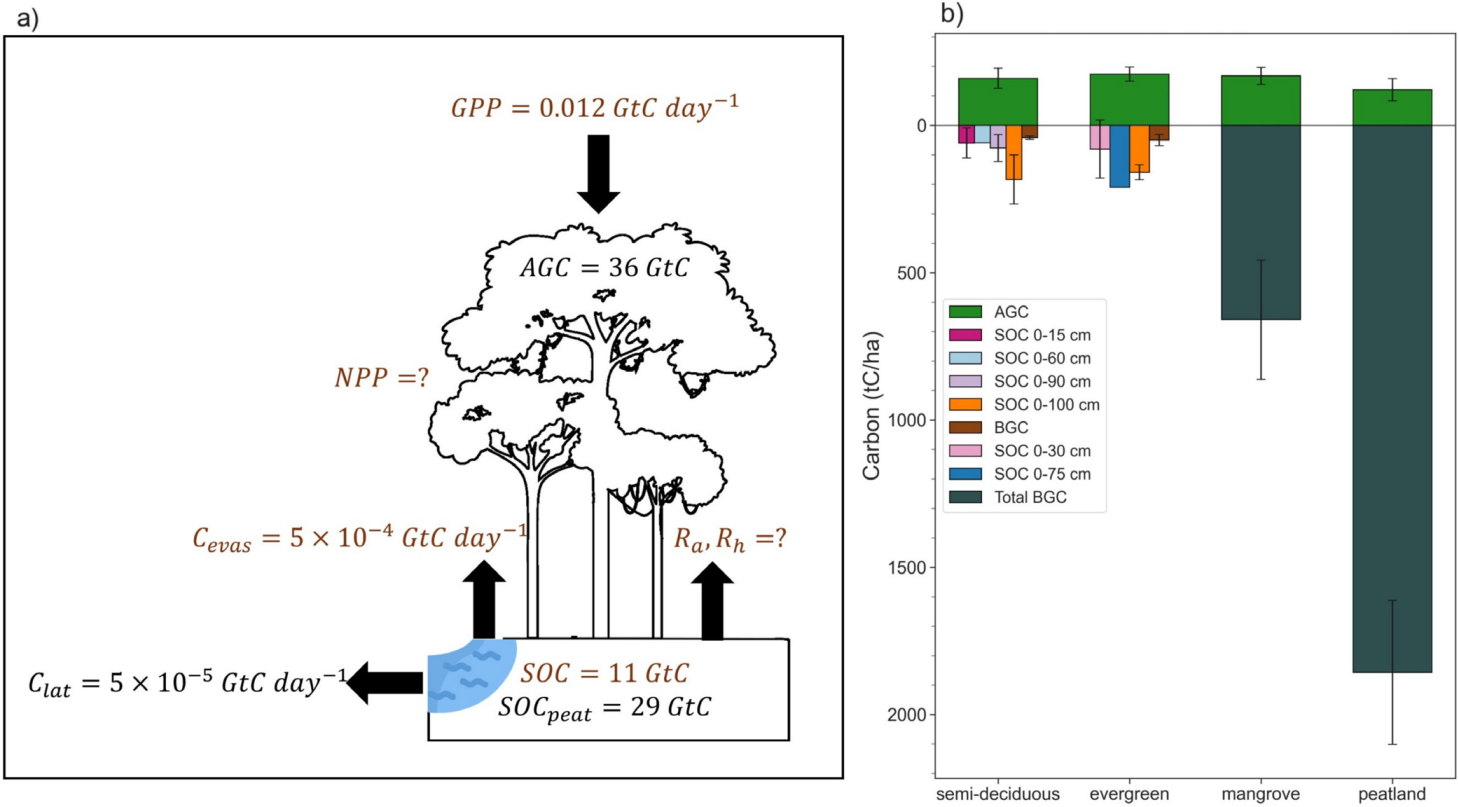
(a) A rough budget of carbon stocks and fluxes in Congo Basin forests. Question marks with NPP, $R_{a}$, and $R_{h}$ indicate they are the most uncertain: “low evidence, low agreement” (Section 2). Carbon evasion ($C_{\text{evas}}$), lateral (riverine) carbon flow ($C_{\mathrm{lat}}=\mathrm{POC}+\mathrm{DOC}+\mathrm{DIC}$), and soil organic carbon (SOC; $\mathrm{SO}C_{\text{peat}}$ represents that of the Cuvette Centrale peatland) are defined in Table 1; (b) average carbon stock density aboveground and belowground for the four main forest types discussed in this study: Semi‐deciduous, evergreen, mangrove, and peatland (Table S1; Bocko et al. 2017; Dargie et al. 2017; Crezee et al. 2022). SOC is reported at different depths, and total BGC is all carbon belowground, living and dead.

At a larger scale, aboveground live biomass is typically estimated by combining airborne or spaceborne laser measurements of forest canopy height, with multi‐source satellite imagery, other geospatial datasets, and plot data (Xu et al. 2017, 2021; Fatoyinbo et al. 2021; Potapov et al. 2021; Saatchi et al. 2011; Sagang et al. 2024; Avitabile et al. 2016; Tyukavina et al. 2013; Baccini et al. 2012; Chen et al. 2015; Kearsley et al. 2013). To provide the rough AGC budget in Figure 3a, we use the estimate provided by Xu et al. (2021) for Central African moist rainforests. Belowground live biomass is calculated from these estimates based on root‐to‐shoot ratios (e.g., Xu et al. 2021). Soil organic carbon (SOC) estimates are typically produced by upscaling soil profiles via statistical or semi‐mechanistic modeling (Hiederer and Köchy 2011; Beillouin et al. 2023; Padarian et al. 2022). To provide the rough SOC budget in Figure 3a, we use the FAO and ITPS Global Soil Organic Carbon Map v1.5, based on in situ measurements between 0 and 30 cm collected between the 1950s and 2000s. We calculate the total ha in SOC that overlaps with $\mathrm{AGC}>120\;\mathrm{tC}\;ha^{-1}.$

Large‐scale estimates of GPP are typically estimated using light‐use efficiency models, solar‐induced fluorescence measurements, or some combination of both (e.g., Madani et al. 2020; Sun et al. 2018). To provide the rough GPP budget in Figure 3a, we use GPP from Madani et al. (2020) and estimate the total ha in GPP estimates that overlaps with $\mathrm{AGC}>120\;\mathrm{tC}\;ha^{-1}.$ Large‐scale estimates of respiration are typically produced by combining plot‐level data and environmental variables using statistical or machine learning methods (Adachi et al. 2017; Tang et al. 2020; Zhao, Ding, et al. 2024).

We prescribe uncertainties of these measurements according to the IPCC 5th Assessment Report guidance note on consistent treatment of uncertainties (Mastrandrea et al. 2011): “Use the following dimensions to evaluate the validity of these measurements: the type, amount, quality, and consistency of evidence (summary terms: “limited,” “medium,” or “robust” evidence), and the degree of agreement (summary terms: “low,” “medium,” or “high” agreement).” Note that these categories are based on a comparison between carbon stock and flux measurements (at both in situ and ecosystem scales) within the region. If we were to apply this categorization to measurements within the Congo Basin compared to the rest of the world, all would be classified as “limited” evidence and “low” agreement due to relative lack of ground‐based measurements and low‐spatiotemporal agreement. Table 1 summarizes these uncertainties.

### 2.1 Current Stocks/Fluxes

**FIGURE 4:**
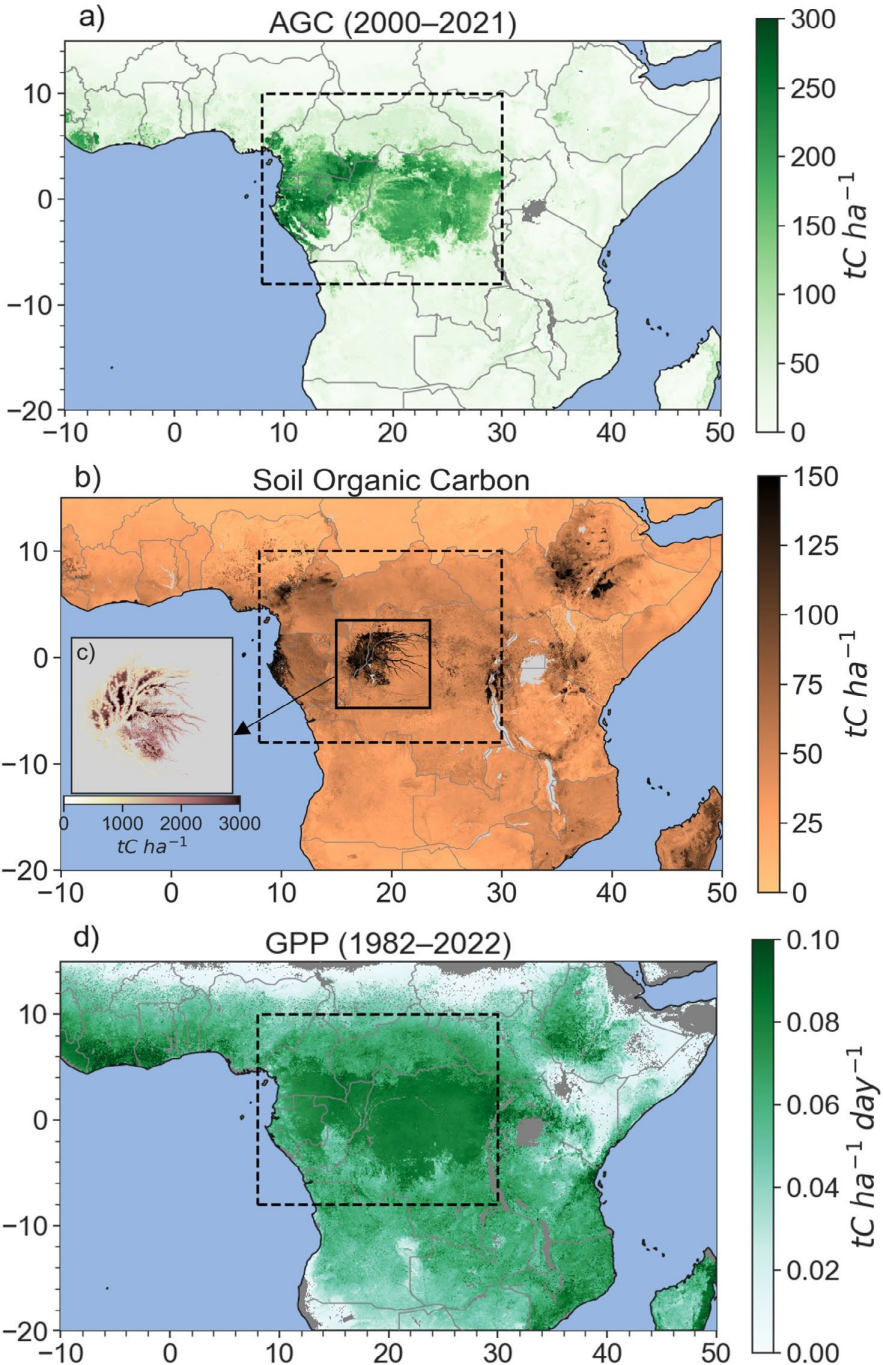
(a) Aboveground biomass (AGC) density averaged between 2000 and 2021 (Xu et al. 2021); (b) soil organic carbon (SOC) density between 0 and 30 cm (GSOCmap; FAO and ITPS 2020); (c) belowground peat carbon density (Crezee et al. 2022; data from https://congopeat.net/maps/); and (d) gross primary productivity (GPP) between 1982 and 2022 (Madani et al. 2020). Map lines delineate study areas and do not necessarily depict accepted national boundaries.

Figure 3 summarizes the mean carbon stocks and fluxes across Congo Basin forests, as well as the mean carbon stock density across vegetation types at the in situ scale (Table S1). Site‐based studies note that intact forests store large aboveground carbon stocks (149–166 tons of C (tC) ha^−1^) (Poulsen et al. 2020; Cuni‐Sanchez et al. 2021; Figure 3b; Table S2), while the Cuvette Centrale peatlands and coastal mangroves store more BGC than AGC: peatlands store 95–157 tC ha^−1^aboveground and 1712 tC ha^−1^ belowground, while mangroves store 136–180 tC ha^−1^ aboveground and 392–967 tC ha^−1^ belowground (Figure 3b; Table S2; Crezee et al. 2022; Kauffman and Bhomia 2017; Ajonina et al. 2014; Bocko et al. 2017; Biddulph et al. 2021). While SOC measurements are limited, one study reports 26 tC ha^−1^ between 0 and 10 cm depth, 108 tC ha^−1^ between 0 and 100 cm depth, and 129 tC ha^−1^ between 0 and 200 cm depth (Schwartz and Namri 2002; Figure 3b). The spatial distribution of AGC, GPP, and SOC are highly heterogeneous at the ecosystem scale (Figure 4).

We found no current ground‐based estimates of NPP or GPP, although this may change with the establishment of the CongoFlux tower in the Yangambi Research Center (Sibret et al. 2022). Limited site‐scale measurements of soil respiration average 11–16.5 tC ha^−1^ yr.^−1^ (Daelman et al. 2025; Baumgartner et al. 2020; Ceulemans 2024). Laterally, the Congo River exports 2 megatons of C (MtC) yr.^−1^ of particulate organic carbon (POC), 12 MtC yr.^−1^ of dissolved organic carbon (DOC), and 4 MtC yr.^−1^ of dissolved inorganic carbon (DIC), while outgassing between 133 and 251 MtC yr.^−1^ of carbon to the atmosphere (Seyler et al. 2006; Coynel et al. 2005; Drake et al. 2018; Spencer et al. 2016; Wang, Bienvenu, et al. 2013; Aufdenkampe et al. 2011; Raymond et al. 2013; Borges et al. 2015, 2019; Table S3). In Figure 3a, $C_{\mathrm{lat}}$ represents the sum of DOC,POC, and DIC from the Congo River, which incorporates areas outside of this study's domain; however, most dissolved carbon comes from the forested areas (Drake et al. 2023). $C_{\text{evas}}$ is the estimated carbon flux evasion from the Congo River.

### 2.2 The Four Key Drivers of Congo Basin Carbon Cycle Change

Table 2 summarizes the four key drivers of Congo Basin carbon cycle changes reviewed in this paper.

**TABLE 2:** A brief description of the four drivers of Congo Basin carbon cycle changes, and topics related to these drivers, reviewed in this paper.

Driver	Topics reviewed
*Climate change*: Altered temperature and precipitation regimes that influence forest carbon cycling by affecting tree growth (↑↓), mortality (↑), and decomposition rates (↑)	Long‐term climatic changes Short‐term droughts El Nino Southern Oscillation (ENSO)
*Land cover and land use changes (LCLUC)*: Changes to land cover including deforestation, forest degradation, forest regrowth, other transition between land cover types, and related land use activities (e.g., figure and agriculture)	Deforestation and forest degradation Biomass burning Animal–ecosystem interactions Nutrient deposition from biomass burning
*CO*_*2*_ *fertilization*: Elevated atmospheric CO_2_ that can boost gross primary productivity through increased water use efficiency	Long‐term increases in atmospheric CO_2_
*Legacy effects*: A post‐event effect on an ecosystem that persists for a long time (e.g., years to millennia) after the initial event has occurred	Past human disturbance and climate change

*Note:* Arrows in the climate change description refer to hypothetical carbon cycle responses of these processes to a changing climate.

## 3 Climate Change

### 3.1 Long‐Term Climatic Changes

**FIGURE 5:**
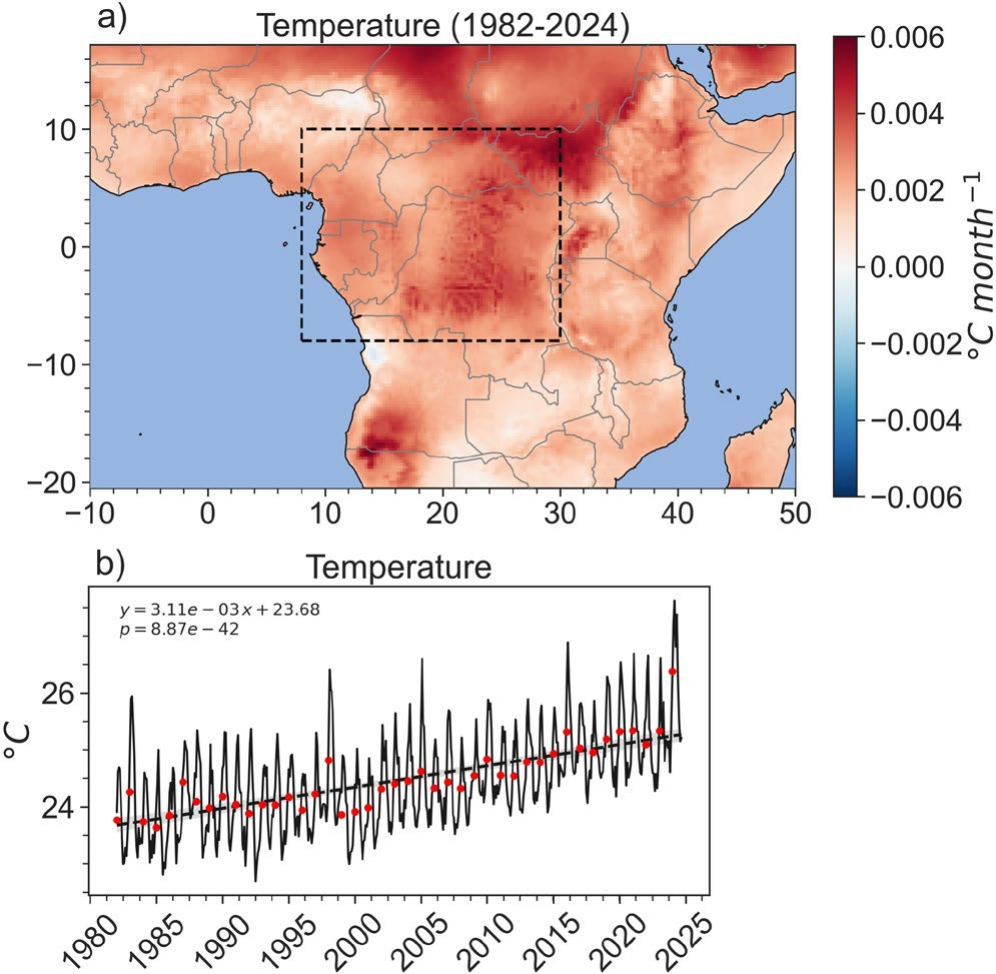
(a) Slope of trend lines of 2 m temperature (the air temperature measured 2 m above the surface of the earth) between 1982 and 2024. Data source: ERA5 Land (Hersbach et al. 2020); (b) time series of 2 m temperature in the Congo Basin forests (filtered by $\mathrm{AGC}>120\;\mathrm{tC}\;ha^{-1}$). Black dashed line indicates the trend (equation and *p*‐value labeled in the plot), while red dots indicate mean annual temperature (MAT) for each year. Map lines delineate study areas and do not necessarily depict accepted national boundaries.

The Congo Basin has experienced long‐term increases in ***temperature***, observed at multiple spatial scales (Alsdorf et al. 2016; Samba et al. 2008; Kazadi and Kaoru 1996; Sonwa et al. 2020; Burnett et al. 2020; Saatchi et al. 2021; Bush, Jeffery, et al. 2020; Kasongo Yakusu et al. 2023; Likoko et al. 2019; Posite et al. 2024; Chapman et al. 2021; Figure 5). Limited studies indicate that increasing temperatures reduce forest growth, subsequently decreasing GPP and AGC in the region (Battipaglia et al. 2015; Hubau et al. 2020; Lewis et al. 2013; Slik et al. 2013). A site‐scale study in Cameroon found that declines in tree growth, measured by tree‐ring analyses, were correlated with local temperature increases (Battipaglia et al. 2015). Additionally, a statistical modeling study, combining inventory plots with temperature, drought intensity, and atmospheric $CO_{2}$ measurements, found that long‐term increases in mean annual temperature reduced carbon gains in the region; however, the Congo Basin remains a carbon sink (Hubau et al. 2020).

While no study examining the impacts of long‐term increases of air or soil temperature on belowground carbon stocks and fluxes in the Congo Basin region exists (e.g., Protti Sánchez et al. 2024), limited studies in other regions or at the global scale have found positive relationships between short‐term increases or seasonal changes in temperature and respiration increases (Okello et al. 2023; Daelman et al. 2025; Liu et al. 2017; Nottingham et al. 2020; Chen et al. 2014; Hursh et al. 2017). This suggests possible increases in soil respiration under long‐term increased temperatures (García‐Palacios et al. 2021; Garcin et al. 2022; Davidson and Janssens 2006), to levels similar to those reported for tropical soils in Central America (Nottingham et al. 2020).

**FIGURE 6:**
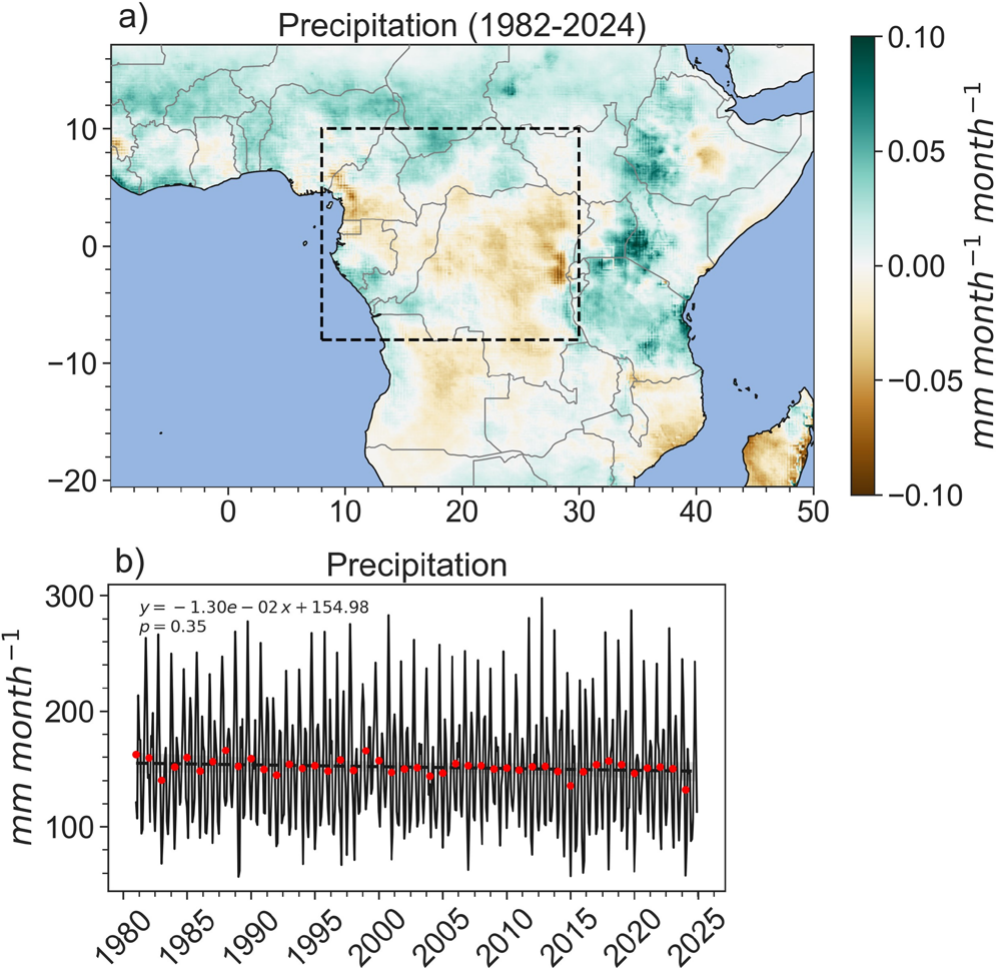
(a) Slopes of monthly precipitation (Funk et al. 2015) linear trend lines between 1982 and 2024 depicting the change in precipitation over time; and (b) time series of monthly precipitation in the Congo Basin forests (filtered by AGC>120 t $C\;ha^{-1}$). Black dashed line indicates the trend (equation and *p*‐value labeled in the plot), while red dots indicate mean annual precipitation (MAP) for each year. Map lines delineate study areas and do not necessarily depict accepted national boundaries.

Multiple studies indicate long‐term declines in precipitation between April–June (Zhou et al. 2014; Nicholson et al. 2022), and increasing precipitation seasonality (i.e., longer dry seasons, drier dry seasons, or wetter rainy seasons) (Kasongo Yakusu et al. 2023; Jiang et al. 2019; Bush, Jeffery, et al. 2020). Long‐term changes in mean annual ***precipitation*** appear more limited across spatial scales (Battipaglia et al. 2015; Kasongo Yakusu et al. 2023; Bush, Jeffery, et al. 2020; Chapman et al. 2021; Figure 6), although many studies acknowledge the paucity of spatially and temporally distributed weather and hydrological stations to validate this lack of trend (Tshimanga et al. 2022; Nicholson et al. 2018).

Few studies assess the impacts of long‐term precipitation changes on Congo Basin carbon stocks and fluxes, with previous studies focusing on the drier West African forests (Fauset et al. 2012; Aguirre‐Gutiérrez et al. 2019, 2020). Isotope data collected from the Yoko Reserve (DRC) between 1958 and 2010 indicates a limited relationship between precipitation and photosynthesis (i.e., GPP) (Colombaroli et al. 2016). Similarly, radial growth was not correlated with long‐term annual variation of rainfall in evergreen understory vegetation in the Luki forest (DRC), although growth appeared to depend on rainfall seasonality (Couralet et al. 2010). Finally, a study based in Libongo, Cameroon, examined the relationship between decreasing tree growth and atmospheric $CO_{2}$, precipitation, and temperature, and found no significant relationship with mean annual precipitation (Battipaglia et al. 2015).

### 3.2 Short‐Term Droughts

**FIGURE 7:**
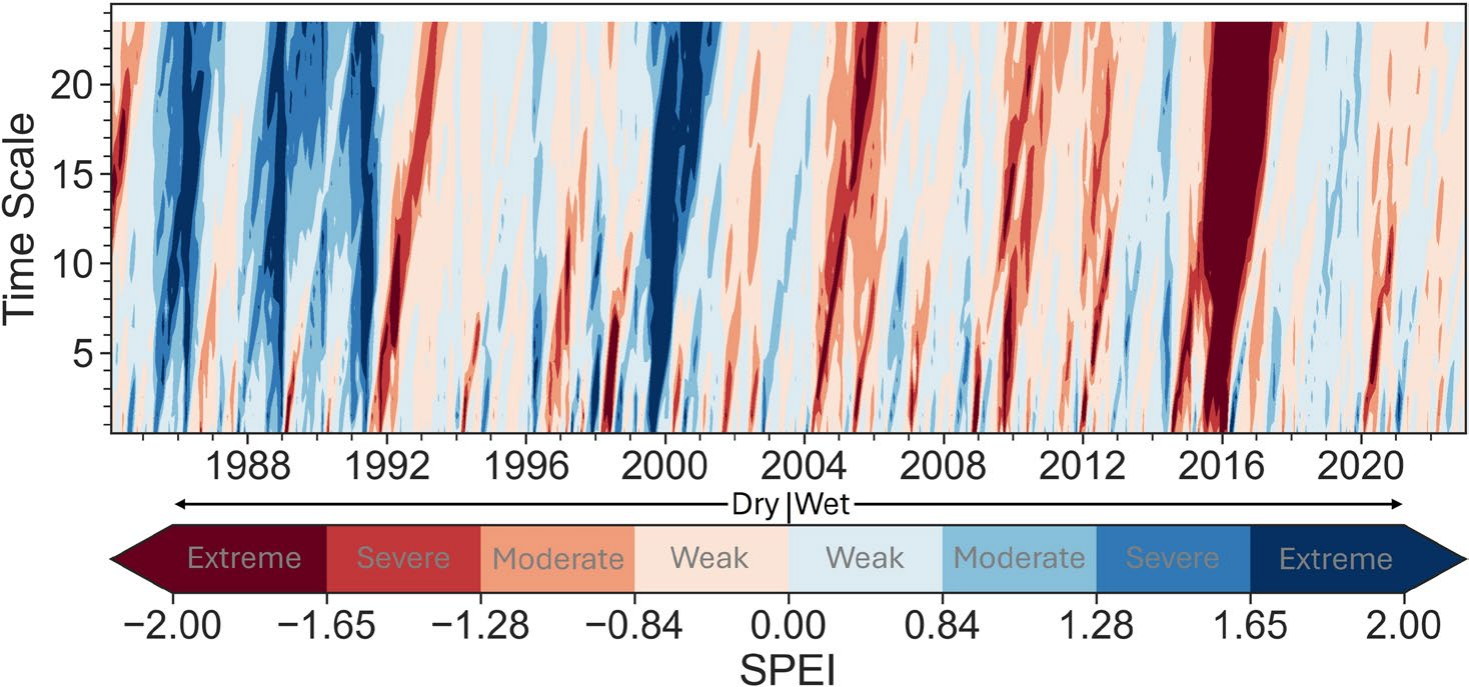
Standardized Precipitation‐Evapotranspiration Index (SPEI) for the Congo Basin forests (filtered by AGC > 120 tC ha^−1^ within the pre‐defined domain). Precipitation dataset used in this calculation comes from CHIRPS (Funk et al. 2015), and potential evapotranspiration used in this calculation comes from GLEAM v3.8a (Martens et al. 2017). SPEI was calculated using the python SPEI package (Vonk 2024). SPEI values between 0 and −0.84 indicate weak drought, between −0.84 and −1.28 indicate moderate drought, between −1.28 and −1.65 indicate severe drought, and less than −1.65 indicates extreme drought (Sorí et al. 2022; Agnew 2000). Similarly, positive values indicate the same scale but for humid periods. Time scale represents the monthly SPEI. For example, SPEI1 indicates meteorological drought, while SPEI12 represents long‐term drought (Sorí et al. 2022).

Short‐term ***drought*** frequency, intensity, and extent have increased across Africa over the last 50 years (Sorí et al. 2022; Spinoni et al. 2014; Cook et al. 2020; Masih et al. 2014; Figure 7). Large swaths of Congo Basin forests display heightened vegetation sensitivity to drought, evidenced by declines in vegetation water content derived from satellite microwave data (Tao et al. 2022; Anderegg et al. 2020). Post drought, GPP typically returns to pre‐drought levels between 6 months and 2 years (Schwalm et al. 2017).

However, Congo Basin forest drought responses are variable. For example, under climatological drought conditions at the M'Baïki experimental site in the CAR (Ouédraogo et al. 2013), tree growth decreased, implying GPP reductions. However, slow‐growing trees experienced higher drought tolerance than fast‐growing trees. Additionally, measurements of canopy backscatter indicate that fragmented landscapes in the northern Congo Basin have displayed a stronger response to short‐term, severe droughts compared to intact forests in the central and eastern part of the region (Asefi‐Najafabady and Saatchi 2013).

### 3.3 El Niño Southern Oscillation (ENSO)

The El Niño Southern Oscillation (ENSO) strongly impacts interannual variations in the tropical region carbon cycle. For example, during El Niño (La Niña) events, tropical vegetation productivity, including within the Congo Basin, is often reduced (increased) due to increases (decreases) in surface temperatures, vapor pressure deficit (VPD), and soil water stress, along with reduced (increased) precipitation (Kim et al. 2017; Liu et al. 2017; Rifai et al. 2019; Dou et al. 2024; Gu and Adler 2011; Ciais et al. 2009). Additionally, one study, using satellite‐based respiration observations across the tropics, found increased vegetation respiration during El Niño years compared to La Niña years (Wang et al. 2022). Modeling‐based studies generally agree with these responses, showing increased atmospheric $CO_{2}$ growth rates driven by reduced NPP during El Niño years, to which tropical regions contribute disproportionally (Kim et al. 2016; Wang, Ciais, et al. 2013; Du et al. 2025). However, spatial variability in carbon cycle response both across the tropics and within the Congo Basin exists in response to ENSO events. To illustrate this spatial variability, we focus on the 2015–2016 extreme El Niño (Santoso et al. 2017), which is relatively well studied within the region compared to past ENSO events.

The 2015–2016 El Niño, which, combined with pre‐existing tropical warming (Rifai et al. 2019), caused severe drought across the tropics, including the Congo Basin (Figure 7b). Using satellite‐based vegetation optical depth measurements, Wigneron et al. (2020) showed that AGC declined by 0.9 GtC in 2014–2016 across forests, shrublands, savannas, grasslands, and croplands in tropical Africa (20° N–20° S). Of these different vegetation types, forest losses within Central Africa contributed the most to this decline, primarily concentrated in Congo Basin countries, but also in Ghana, Côte d'Ivoire, and Uganda (Wigneron et al. 2020).

After this El Niño event, total AGC did not recover to pre‐drought levels in 2017, primarily due to further declines in forest AGC that countered recovery in the shrubland and savanna regions (Wigneron et al. 2020). This is partially spatially consistent with more negative solar‐induced fluorescence (SIF, a proxy for GPP) anomalies in 2016 (Luo et al. 2018). However, these further declines were concentrated in Uganda and the DRC, while the other regions regained AGC (Wigneron et al. 2020). The starting year of AGC recovery for the Congo Basin forests varied between 2015 and 2018 (Yang et al. 2022). Recovery time was heterogeneous within evergreen broadleaf forests: the upper canopy layer and leaves typically recovered within 1–4 months, while 42% of the woody components took over 12 months to recover (Dou et al. 2024). This differs from much more rapid recovery (2 months) of woody components in South American evergreen broadleaf forests (Dou et al. 2024). This is attributed to continental differences in woody recovery times to stronger drought impacts, less favorable moisture conditions, and larger temperature and precipitation monthly standard deviations in tropical Africa forest regions (Dou et al. 2024).

Over a larger region, studies using inverse modeling and analyses of atmospheric $CO_{2}$ showed that tropical Africa released 0.5–1.65 GtC during the 2015/2016 El Niño (Liu et al. 2017; Gloor et al. 2018; Palmer et al. 2019), which was attributed to increased respiration under high‐temperature anomalies (Liu et al. 2017). However, these studies also indicate that much of the western and central parts of tropical Africa did not release $CO_{2}$ at significantly higher rates compared to pre‐El Niño conditions. Instead, areas with significant carbon release were in western Ethiopia, western Africa (Côte d'Ivoire, Ghana, Togo, Benin, and Nigeria), and some parts of southern tropical Africa (Angola, southern DRC, and Zambia), which include areas with large SOC stores and substantial land use change (Palmer et al. 2019; Liu et al. 2017).

The results of the inverse modeling studies appear to align with a ground‐based study using 100 long‐term forest inventory plots in West (Ghana and Liberia) and Central Africa (Cameroon, Gabon, Equatorial Guinea, RoC, and DRC). Results indicated limited (not statistically significant) reductions in the live biomass carbon sink, linked to increased drought conditions during the 2015/2016 E; Niño (Bennett et al. 2021). Notably, these forest plots remained a net carbon sink at 0.51 ± 0.40 tC ha^−1^ yr.^−1^. These plots were primarily located in areas that overlap with weak carbon emissions in Liu et al. (2017) and Palmer et al. (2019), suggesting resilience in these areas; although the spatial heterogeneity at a larger scale, as indicated by Wigneron et al. (2020), remains an important and poorly understood part of the story.

## 4 Land Use and Land Cover Changes

### 4.1 Deforestation and Forest Degradation

**FIGURE 8:**
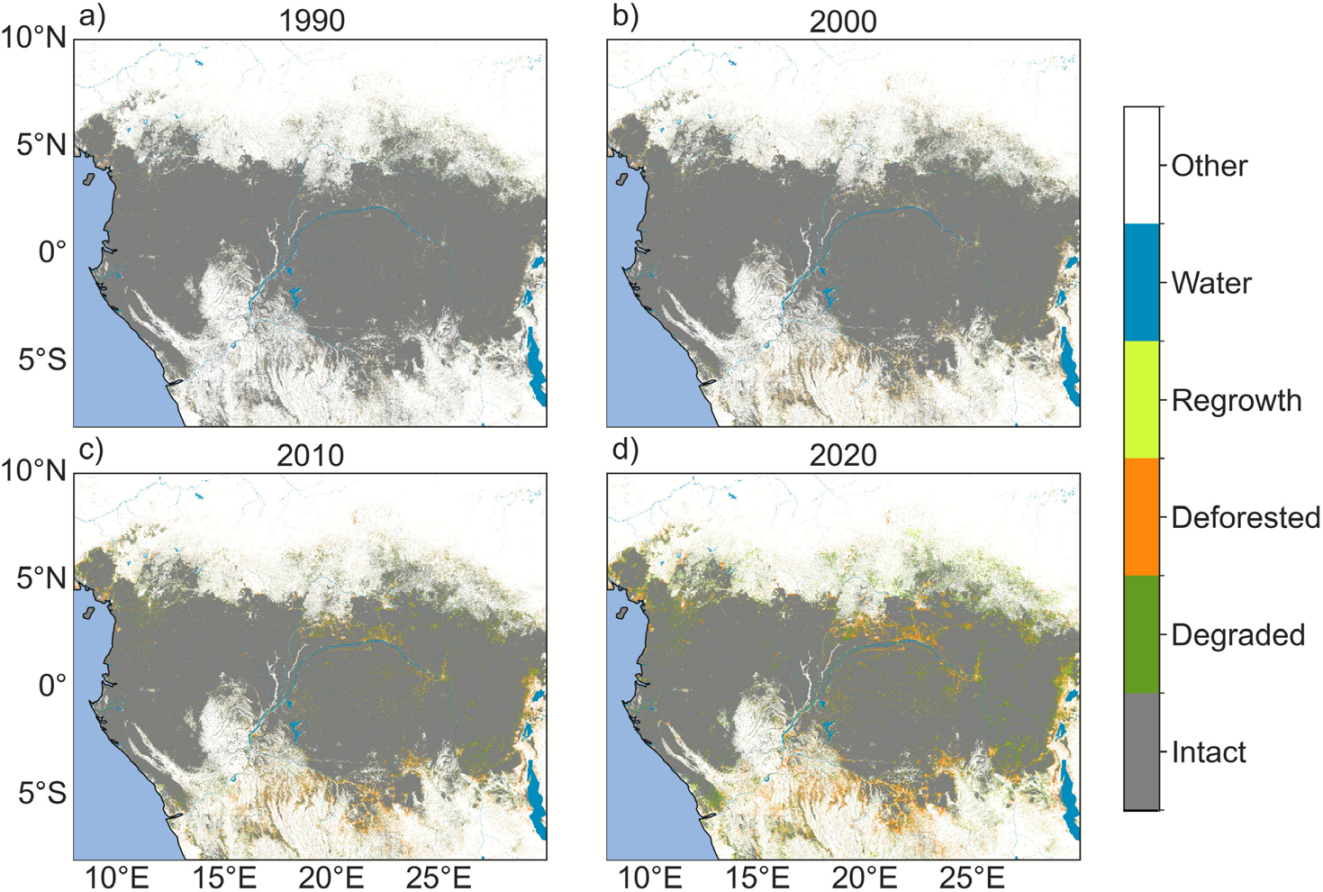
Annual forest change from the Tropical Forest Monitoring Project (*Source:* EC JRC; Vancutsem et al. 2021). For (a) 1990; (b) 2000; (c) 2010; and (d) 2020.

The Congo Basin has historically experienced less deforestation (permanent conversion of forest to another vegetation type) and forest degradation (here defined as a transitory or long‐term decline in forest conditions; Lapola et al. 2023, but see Vancutsem et al. 2021 regarding degraded forest classifications from satellite observations) than other tropical regions (Vancutsem et al. 2021; Rosa et al. 2016). However, deforestation (1990–1999: 0.5 Mha, 2010–2019: 0.9 Mha) and degradation (1990–1999: 0.6 Mha, 2010–2019: 1.1 Mha; Vancutsem et al. 2021) have risen since the 1990s (Tyukavina et al. 2018; Vancutsem et al. 2021; Hufkens et al. 2020; Turubanova et al. 2018; Feng et al. 2022; Kleinschroth et al. 2019; Kleinschroth and Healey 2017; Shapiro et al. 2023; Figure 8). Over 80% of clearing is due to shifting subsistence and commercial agriculture (Molinario et al. 2015; Kotto‐Same et al. 2000; Shapiro et al. 2023; Tyukavina et al. 2018; Mangaza et al. 2022; Masolele et al. 2024). Other important and growing sources of disturbance include logging, mining, agro‐industry, and the expansion of road systems to support these industries (Biswas et al. 2025; Tchatchou et al. 2015; Kleinschroth et al. 2019; Ladewig et al. 2024; Feintrenie 2014; Tyukavina et al. 2018). Roughly 30% of the region's forests lie within logging concessions (Laporte et al. 2007; Eba'a Atyi et al. 2022), although coverage varies (Chervier et al. 2024): 70% of Gabon consists of logging concessions and industrial agriculture (Sagang et al. 2024), versus 10% in the DRC (Chervier et al. 2024; Bayol et al. 2022). This is exemplified by the large and increasing number of logging roads within the western Congo Basin compared to the interior; between 2003 and 2018, road length inside logging concessions doubled (Kleinschroth et al. 2015, 2019).

Deforestation and forest degradation directly reduce AGC in the Congo Basin (e.g., Ngueguim et al. 2009; Dupuis et al. 2024; Medjibe et al. 2011; Mokake et al. 2024; Poulsen et al. 2013; Ajonina et al. 2014; Kotto‐Same et al. 1997; Cazzolla Gatti et al. 2015; Makelele et al. 2021; Sullivan et al. 2022; Besisa Nguba et al. 2025). Conversion to agriculture can result in 50%–99% losses of AGC depending on the type of cropland (Kotto‐Same et al. 1997; Mangaza et al. 2022). Logging impacts on AGC vary with the type and level of management. Selectively logged sites in Gabon, Cameroon, and the RoC show ~8% AGB decline immediately post‐harvest, from 420–435 to 385–401 t ha^−1^ (Dupuis et al. 2024; Medjibe et al. 2011). However, across all of Gabon, forests in logging concessions show larger AGC compared to intact forests, likely due to selective harvest of high‐value, carbon‐dense timber species and increased productivity (Sagang et al. 2024).

AGB recovery post‐disturbance depends on disturbance type, soil nutrients, erosion vulnerability, and invasive species (Kleinschroth et al. 2015; Kleinschroth and Healey 2017; Djiofack et al. 2024). Abandoned croplands have been shown to recover only 8% of their biomass within 5–10 years, 39% after 20–40 years, and 43% after 60 years (Mangaza et al. 2022; Makelele et al. 2021). Abandoned logging roads recover only about 6% of nearby forest biomass after 15–30 years (Kleinschroth et al. 2016). These biomass reductions may persist for hundreds of years (Bauters, Vercleyen, et al. 2019).

Additionally, these disturbances alter tree species diversity and function (e.g., Bauters, Vercleyen, et al. 2019; Amani et al. 2021, 2022), and can decrease essential soil nutrients, compromising mature tree growth and shifting the functional composition of regenerating vegetation (Bauters, Moonen, et al. 2021; Bauters et al. 2016; Teutscherova et al. 2022). While species richness and diversity can recover within 30–60 years post‐abandonment, secondary forest (20–40 years) composition and structure are fundamentally altered compared to mature forest (Mangaza et al. 2022; Depecker et al. 2022; Bauters et al. 2016; Bauters, Moonen, et al. 2021; Kleinschroth et al. 2016). Even in low‐intensity, selectively logged sites, species composition differs, particularly in the understory (Sullivan et al. 2024), likely due to increased canopy gaps favoring the growth of light‐demanding species.

These disturbance and recovery patterns can induce changes to the biogeochemical and hydrological cycles, such as increased runoff, flooding frequency and severity, and erosion, all of which can alter lateral carbon fluxes (Drake et al. 2019, 2020, 2024; Bradshaw et al. 2007; Richey et al. 2022). For example, a stream nearby shifting agriculture near Kisangani, DRC, showed higher carbon export and streamflow than an intact forest stream (Drake et al. 2024).

Belowground, these disturbances can impact SOC and respiration. Lower SOC has been observed in selectively logged areas compared to unlogged forests (Chiti et al. 2016; Lontsi et al. 2019), with declines persisting up to 50 years (Chiti et al. 2016), likely due to organic matter removal during logging and absence of plant material inputs after logging (Lontsi et al. 2019). Agriculture further decreases SOC through increased streamflow and erosion, although agroforestry systems, especially those with nitrogen‐fixing tree species, can mitigate some of these effects (Koutika et al. 2021; Mandah et al. 2024). Furthermore, in selective reduced‐impact logging areas in Cameroon, measurements indicate a 28% reduction in soil $CO_{2}$ emissions (i.e., soil respiration) compared to intact forests (Lontsi et al. 2020). In contrast to many studies, measurements taken in Cameroon indicate that soil respiration in agroforestry and cropland regions is lower compared to secondary forests (Verchot et al. 2020).

### 4.2 Biomass Burning

**FIGURE 9:**
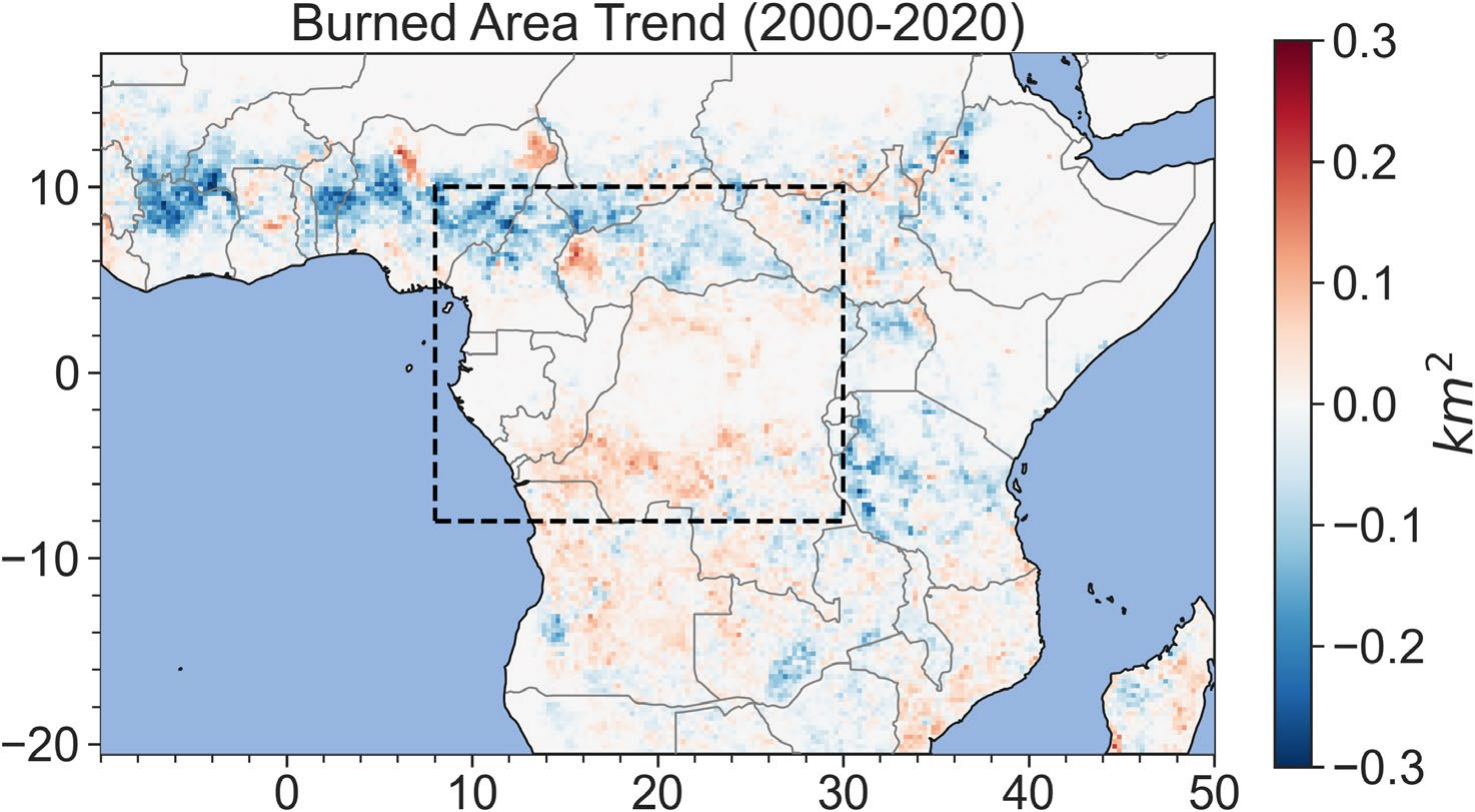
Slope of the linear trendline of burned area, representing change in burned area (km^2^), between 2000 and 2020 from GFED5 (Chen et al. 2023b). Map lines delineate study areas and do not necessarily depict accepted national boundaries.

Only 2%–3% of total burned area in Africa, due to small (< 100 ha) or large fires (> 100 ha), is found in forests (Ramo et al. 2021; Figure 9). Most fires in the Congo Basin remain quite small, resulting from slash‐and‐burn agriculture (Van Der Werf et al. 2017; Andela et al. 2017; Archibald et al. 2012). Furthermore, many global burned‐area datasets have coarse (250–500 m) resolution; therefore, small agricultural fires are likely undetected or underestimated (Chuvieco et al. 2022; Ramo et al. 2021). However, it is important to detect small fires as they reduce AGC and BGC (Kotto‐Same et al. 1997; Tabi et al. 2013; Mandah et al. 2024), alter forest composition (Cirezi et al. 2022) and nutrient availability (Njomgang et al. 2006), and create new forest edges that can exacerbate carbon losses via localized increases in canopy dryness (Zhao et al. 2021). For example, the carbon deficit from edge effects due to fire is estimated to be 3.8 tC ha^−1^ in all African moist forests and 6.4 GtC ha^−1^ in all African dry forests (Zhao et al. 2021).

### 4.3 Plant–Animal Interactions

**FIGURE 10:**
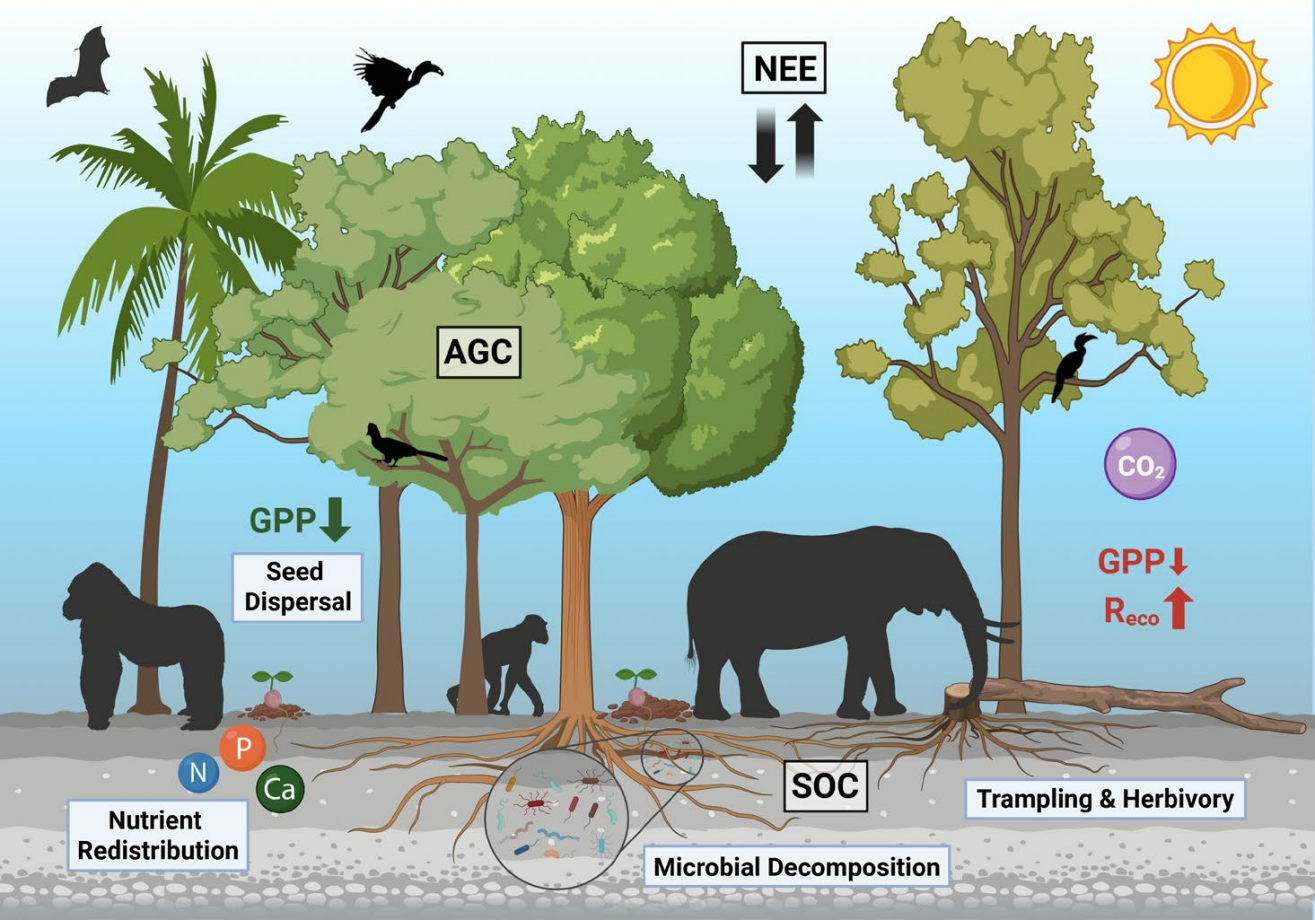
Plant–animal interactions and their influence on carbon cycling in the Congo Basin, building on Schmitz et al. (2018). Green indicates carbon uptake, while red indicates carbon losses. AGC, aboveground carbon; Ca, calcium; N, nitrogen; NEE, net ecosystem exchange; P, phosphorus; R_eco_, ecosystem respiration (including autotrophic and heterotrophic respiration); SOC, soil organic carbon.

Animals impact carbon cycling by distributing carbon and other nutrients in dung and carcasses, dispersing seeds of carbon‐dense trees, and influencing tree growth and forest structure via herbivory and trampling (e.g., Malhi et al. 2022; Schmitz et al. 2018; Figure 10). Overhunting and habitat fragmentation threaten the key ecosystem services contributed by animals in the Congo Basin (Blake et al. 2009; Poulsen et al. 2013; Poulsen et al. 2017; Bush, Whytock, et al. 2020; Ingram et al. 2025).

Megafauna play a disproportionate role in carbon cycling of Congo Basin forests compared to other animals owing to their size, energetic contributions, and seed dispersal capacity. African forest elephants ( *Loxodonta cyclotis* ), the largest animals in these ecosystems, can disperse the greatest diversity of intact seeds over the longest distances–at least tens of kilometers (Blake et al. 2009; Campos‐Arceiz and Blake 2011; Poulsen et al. 2021; Bush, Whytock, et al. 2020; Breuer et al. 2016; Poulsen et al. 2017; Maisels et al. 2013). Large‐seeded tree species, some of which rely exclusively on African forest elephants for dispersal over long distances, tend to have the highest wood density and subsequently the greatest carbon sequestration capacity (Deblauwe et al. 2025; Poulsen et al. 2021; Campos‐Arceiz and Blake 2011). One example is West African ebony ( *Diospyros crassiflora* ), with an average wood density of over 1.1 g cm^−3^, which depends on forest elephants for dispersal (Deblauwe et al. 2025). In addition to seed dispersal, elephant trampling and browsing decrease forest stem density, which can alter light and water availability and the spatial partitioning of resources. This promotes the persistence of larger‐statured, high wood density trees, and thus high carbon sequestration (AGB), but may decrease NPP as larger and slower growing trees dominate (Berzaghi et al. 2019, 2022, 2023).

As African forest elephant populations decline, their functional extinction is hypothesized to affect tropical forests in the Congo Basin by shifting plant species composition, increasing stem density, and reducing large tree abundance (Poulsen et al. 2018; Brodie et al. 2025). However, large uncertainties remain in quantifying these impacts, including how they are changing with increasing anthropogenic disturbances.

### 4.4 Nutrient Availability

**FIGURE 11:**
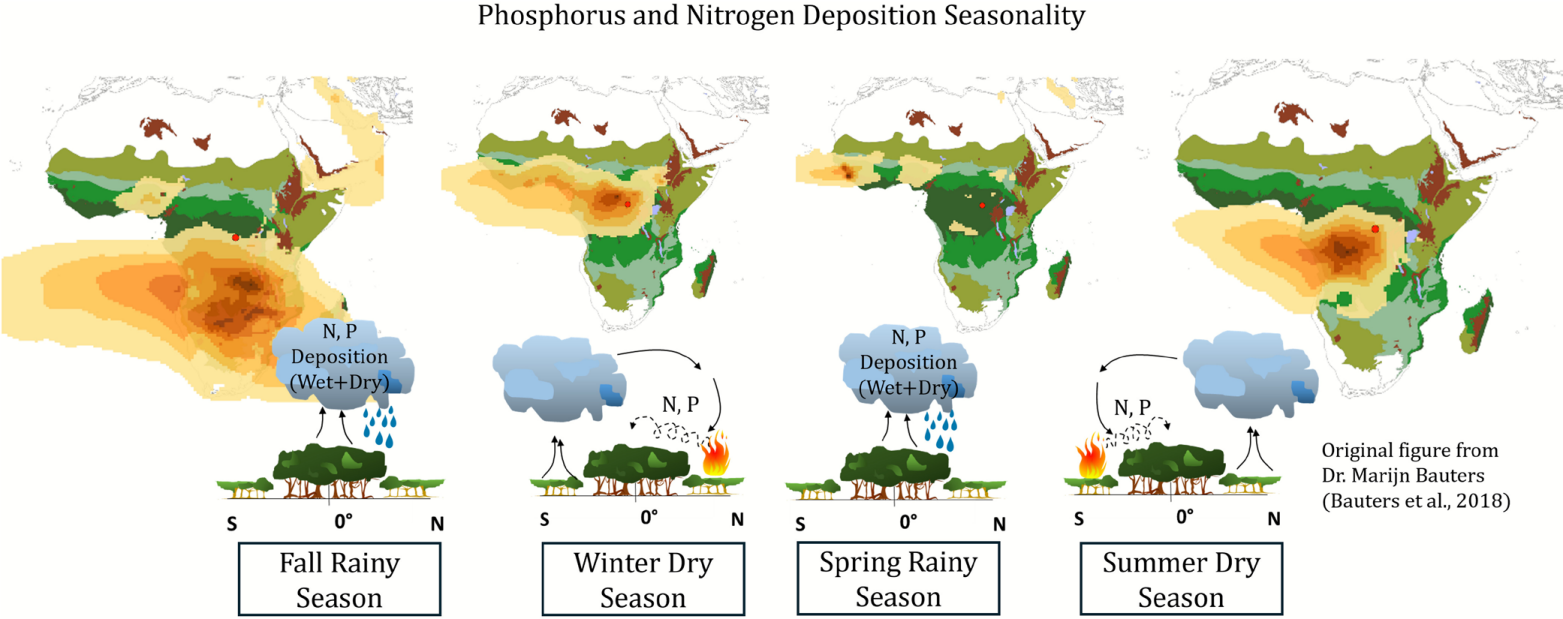
Schematic representation of nutrient and phosphorus deposition into the Congo Basin forests during its two rainy seasons in boreal fall and spring and two dry seasons in boreal winter and summer. Shaded greens represent land cover types, shaded orange contours represent aerosol optical depth, and the red dot represents the location of the site study from Bauters et al. (2018) and Bauters, Drake, et al. (2021). Adapted from Bauters et al. (2018).

Frequent and large‐scale savanna fires north and south of the Congo Basin account for 70% of global burned area and about half of global fire‐related carbon emissions (Andela et al. 2017; Ramo et al. 2021; Archibald 2016). These fires, and dust from the Sahara Desert (Okin et al. 2004; Lamancusa and Wagstrom 2019), supply nutrients to the Congo Basin via wind‐driven smoke and dust plumes (Bauters et al. 2018; Ito et al. 2015; Chen et al. 2010; Goll et al. 2023; Bauters, Drake, et al. 2021; Okin et al. 2004), with subsequent deposition of nitrogen (N) and phosphorus (P) (Bauters et al. 2018; Ito et al. 2015; Chen et al. 2010; Goll et al. 2023; Bauters, Drake, et al. 2021; Figure 11). Both transport and deposition appear seasonal: during the dry seasons, plumes carrying N and P from intense biomass burning to the north and south of the forests are transported into the Congo Basin via cross‐equatorial winds. During the rainy seasons, these nutrients are deposited (Bauters et al. 2018; Bauters, Drake, et al. 2021; Figure 11). In total, Congo Basin forests receive approximately 18.2 kg N ha^−1^ yr.^−1^ as wet deposition (exceeding deposition in Central America, Southeast Asia, and West Africa), with additional dry deposition via canopy interception. Secondary forests can receive up to 3.1 ± 1.4 kg P ha^−1^ yr.^−1^ from wet and dry deposition (Bauters et al. 2018; Bauters, Drake, et al. 2021; Williams et al. 1997; Kohler et al. 2013; Van Langenhove et al. 2020).

Model simulations suggest that P deposition significantly increases AGB sequestration within the Congo Basin (Du et al. 2020; Goll et al. 2023). For example, Goll et al. (2023) used the ORCHIDEE nutrient‐enabled land surface model, calibrated with data from undisturbed, mixed lowland forest sites in the Yangambi and Yoko reserves in the DRC, to examine the impacts of P and other nutrient additions on AGB and GPP. When tripling P inputs, AGB increased by 380% and GPP increased by 13% between 2000 and 2019 compared to the baseline simulation. No significant effect on either AGB or GPP was observed when increasing N inputs alone (Goll et al. 2023).

LCLUC can also alter nutrient cycling, with subsequent carbon cycle impacts. Repeated clearing primarily reduces soil cation stocks, although it can also lead to declines in plant‐available N in the topsoil (Bauters, Moonen, et al. 2021). This is due to the mobility of cations in the soil, and the large proportion of cations stored in aboveground woody biomass (Bauters et al. 2022). In addition, secondary forests cycle higher amounts of N and P deposition via more efficient dry deposition filtering (Bauters, Drake, et al. 2021; Makelele et al. 2022). Despite substantial N and P inputs, research along a successional gradient in the DRC demonstrated persistent cation (e.g., calcium) scarcity as the likely key limiting factor in forest regeneration and subsequent recovery of AGC (Bauters et al. 2022).

## 5 Elevated Carbon Dioxide (CO_2_)

Studies argue that elevated atmospheric $CO_{2}$ concentrations (i.e., $CO_{2}$ fertilization) have accelerated photosynthesis, increased WUE, and subsequently increased the land carbon sink (Ruehr et al. 2023; Walker et al. 2021; Keeling et al. 2017; Schimel et al. 2015; Fernández‐Martínez et al. 2019). However, it is important to note that (1) its impact can vary across plant type, nutrient, light, and water availability regimes, and can be modulated by shifts in carbon allocation; (2) studies consistently note large uncertainties associated with estimation of this effect in the tropics; and (3) long‐term, ground‐based studies and region‐specific models examining the effect of elevated $CO_{2}$ on carbon cycling are extremely scarce within the Congo Basin.

Some studies, using carbon isotopes in tree rings, do not show sustained stimulation of tree growth in response to $CO_{2}$ fertilization in the Congo Basin, but do indicate long‐term increases in intrinsic WUE (iWUE). This indicates an increase in net photosynthesis, a decrease in stomatal conductance, or both (Van Der Sleen et al. 2015; Wils et al. 2016). However, herbarium samples from the DRC reveal a decline in iWUE over the past century, despite reduced stomatal density and increased potential photosynthetic capacity via increases in leaf nutrients (Bauters et al. 2020). This decline may result from rising temperatures, reduced precipitation, and higher VPD, which exacerbate water stress and limit the benefits of $CO_{2}$ fertilization. These findings suggest that the $CO_{2}$ fertilization effect on iWUE in Congo Basin forests is complex and modulated by climate change. Furthermore, changes in iWUE depend on tree height and light availability, suggesting that tree species may differ in their ability to increase iWUE in response to increased atmospheric $CO_{2}$ (Brienen et al. 2017).

## 6 Legacy Effects

Over the past ~20,000 years, the Congo Basin has experienced significant shifts in precipitation and temperature, and anthropogenic disturbance, driving major changes in forest and savanna distributions (e.g., Bonnefille et al. 1990; Weijers et al. 2007; Izumi et al. 2023; Ivory and Russell 2016; Lézine et al. 2019). Here, we focus on the past 5000 years, which likely holds more direct relevance for current vegetation structure and physiology.

During the late Holocene period (after 3000 BP), mature forest in Cameroon, Gabon, and RoC regressed, likely reducing carbon stocks and increasing forest fragmentation and pioneer species (e.g., Ngomanda et al. 2009; Vincens et al. 1999, 2010; Brnčić et al. 2007). This shift is linked to greater rainfall seasonality introducing longer dry periods and/or increasing human disturbance with the arrival of the Bantu people (Vincens et al. 1999, 2010; Ngomanda et al. 2007, 2009; Garcin et al. 2018). Additionally, prolonged drying between 5000 and 2000 cal. yr. BP led to peat decomposition in RoC (Garcin et al. 2022), suggesting a drought threshold for carbon storage. Since ~2000 BP, climate oscillated between wetter and drier periods (Garcin et al. 2022; Brnčić et al. 2007, 2009; Morin‐Rivat et al. 2017; Ngomanda et al. 2005). For example, a wetter period in the late 18th century may have driven evergreen rainforest expansion, reducing light‐demanding species (Maley 2009; Ngomanda et al. 2007; Biwolé et al. 2015). Overall, studies suggest that these repeated climate oscillations influenced vegetation drought adaptations, leading to potentially increased drought tolerance among modern species (Bennett et al. 2021; Asefi‐Najafabady and Saatchi 2013; Parmentier et al. 2007; Luambua et al. 2021). However, ecophysiology studies needed to confirm these evolutionary adaptations among modern plant species remain essentially non‐existent.

Historically, anthropogenic disturbance has also varied, with significant changes in population occurring between 1300 and 1000 BP and with European colonization in the 1800s (Garcin et al. 2022; Brnčić et al. 2007, 2009; Morin‐Rivat et al. 2017). Some studies hypothesize that current Congo Basin vegetation structure, including light‐demanding trees and abundant pioneers, reflects long‐term anthropogenic disturbance, including burning and oil palm and rubber cultivation (Oslisly et al. 2013; Brnčić et al. 2007; Harp 2015), assuming that natural disturbances (e.g., canopy gaps resulting from treefall) are relatively rare events in mature forests (Mueller‐Dombois and Ellenberg 1974; White 1979; Luambua et al. 2024). Within Cameroon, Gabon, and RoC, this hypothesis is supported by dense “pockets” of light‐demanding species that show regeneration deficits and differ significantly from surrounding forests in terms of species composition (Morin‐Rivat et al. 2017; Van Gemerden et al. 2003; Vlam et al. 2017). These “pockets” coincide with the end of intense slash‐and‐burn farming activity in the late 19th century, leaving former farmland to regenerate and introducing these light‐demanding species (Biwolé et al. 2015; Van Gemerden et al. 2003; Ngomanda et al. 2005; Morin‐Rivat et al. 2017; Vleminckx et al. 2014; Bourland et al. 2015; Ligot et al. 2019; Guidosse et al. 2022; Luambua et al. 2021). In more recent years, these species are declining and may reach the end of their successional stages as current disturbance (e.g., canopy openings from logging) may be too small to sustain them (Morin‐Rivat et al. 2017; Biwolé et al. 2015), potentially altering fluxes as these species shift toward shade‐tolerant species.

However, interior Congo Basin forests in the DRC neither meet the human disturbance hypothesis criteria, nor share the same human history. Instead, they appear to be old‐growth ecosystems characterized by natural disturbance‐driven gap dynamics (Luambua et al. 2024). This highlights spatial heterogeneity in the response of Congo Basin vegetation to past human activity, and emphasizes the large uncertainties associated with attributing current vegetation structure and function to past climate and anthropogenic disturbances.

## 7 Driver Interactions

While each driver affects Congo Basin carbon cycling individually, their interactions often form feedback loops—for example, droughts can promote deforestation, which in turn worsens drought impacts. Here, we highlight several examples of the impacts of these interactions on the Congo Basin carbon cycle.

Long‐term climate change is intensifying extreme‐event impacts on Congo Basin carbon cycling. CMIP5 models indicate that rising temperatures have increased GPP sensitivity to El Niño events and droughts, but with strong spatial heterogeneity (Kim et al. 2017; He et al. 2021). With models projecting further rises in temperature, extreme rainfall, drought, and flooding, these impacts are likely to worsen (Karam et al. 2022; Ludwig et al. 2012; Seidou et al. 2025; Aloysius et al. 2016).

Deforestation‐drought interactions are complex, but highly likely given the tightly coupled land‐atmosphere dynamics in the region. Deforestation can exacerbate drought impacts by reducing moisture recycling, and therefore rainfall both locally and downwind (Worden, Fu, et al. 2021; Baker and Spracklen 2022; Sorí et al. 2022; Nyasulu et al. 2024; Van der Ent et al. 2010; Te Wierik et al. 2024), but these interactions remain understudied in the Congo Basin. Deforestation in tropical regions, including the Congo Basin, also increases surface temperature due to changes in the surface energy balance via ET and surface roughness that overtake any cooling impacts from albedo changes (Lawrence et al. 2022; Zeppetello et al. 2020; Smith, Robertson, et al. 2023; Zeng et al. 2021). This further exacerbates long‐term temperature increases due to climate change, with follow‐on effects. In an idealized deforestation simulation, CMIP6 models indicate hotter and drier conditions induced by deforestation amplify carbon losses in the Congo Basin watershed (Li et al. 2022).

Droughts, in turn, can affect LCLUC and biomass burning. While deforestation and degradation are rising, their timing and intensity often align with droughts (Girard et al. 2021). From 2003 to 2021, fires increased near forest edges and the Congo River, areas with high temperature and VPD variability and rapid deforestation (Chen et al. 2023a; Wimberly et al. 2024). Biomass burning increases during the dry season, and combined with seasonally low precipitation, is associated with reduced photosynthesis and increased atmospheric $CO_{2}$ (Jiang et al. 2023).

Some models suggest CO_2_ fertilization may offset carbon losses from climate change and deforestation (e.g., Cao et al. 2001; Ciais et al. 2009; Fisher et al. 2013). For example, Bilir et al. (2025), using a Bayesian land surface model and data integration framework, found net increases in total carbon storage in the Congo Basin (2000–2021) due to rising atmospheric $CO_{2}$ overtaking negative impacts of long‐term climate trends. Dynamic vegetation models similarly identify CO_2_ as the main driver of biomass increases through 2100 (Brandt et al. 2017; Huntingford et al. 2013), while ORCHILEAK simulations link historical rises in NPP and carbon export primarily to CO_2_ fertilization (Hastie et al. 2020).

However, terrestrial biosphere models may overestimate $CO_{2}$ fertilization effects (Pan et al. 2011; Koch et al. 2021) by poorly representing nutrient limitations (especially P; Fleischer et al. 2019), vegetation optimal temperatures (Sibret et al. 2025), ozone impacts on GPP (Brown et al. 2022), and climate‐driven mortality (Gora and Esquivel‐Muelbert 2021). These model limitations raise questions about the accuracy of predicted $CO_{2}$ fertilization effects, particularly as site‐scale, tree‐ring studies suggest limited empirical evidence of plant growth responses to increased $CO_{2}$.

## 8 Recommended Future Research Directions

We recommend several research directions to advance understanding of the impacts of these four drivers of change, and their interactions, on Congo Basin carbon stocks and fluxes. First, large uncertainties in carbon stock and flux measurements impede understanding of responses to these drivers. Past and current efforts to quantify carbon stocks include spaceborne missions such as GEDI, NISAR, and BIOMASS as well as increased field and airborne measurements such as the 2016 AfriSAR campaign in Gabon (Fatoyinbo et al. 2021) and several additional countries in 2024. However, more extensive in situ and airborne measurements are needed, particularly of fluxes (Baumgartner et al. 2020; Barthel et al. 2022) and measurements that enable direct scaling with remote sensing (Ordway et al. 2025). Long‐term in situ monitoring is crucial to better characterize spatiotemporal variability in carbon stocks and fluxes, including decoupling reduced productivity from increased autotrophic and heterotrophic respiration during periods of reduced net carbon uptake.

Furthermore, additional experimental and modeling‐based studies are needed to understand: (Abernethy et al. 2016) how these drivers are changing over time (climate change and extreme event variability, LCLUC intensity, atmospheric $CO_{2}$ growth rates); and (Acobta et al. 2023) how these changes are interacting and therefore creating feedbacks that amplify or dampen carbon cycle responses. Experimental studies might include impacts of nutrient additions, carbon allocation shifts, throughfall exclusion, and fire manipulation on Congo Basin carbon cycling. Such observationally based experiments have been mostly or exclusively performed in other parts of the tropics, or experiments have been conducted using modeling within the Congo Basin (Goll et al. 2023; Wright 2019; Poorter et al. 2012; Vargas Gutiérrez et al. 2023; Deklerck et al. 2019; Higgins et al. 2007). Additionally, multiple kinds of models are essential for providing process‐based explanations of ecological feedbacks (e.g., vegetation‐climate or fire‐carbon feedbacks) that influence whether interacting drivers stabilize or destabilize Congo Basin carbon dynamics. They include global climate models (Anderegg et al. 2022; Luo et al. 2022), regional climate and climate‐vegetation models (Bangelesa et al. 2023; Wang et al. 2016; Fotso‐Nguemo et al. 2019; Akkermans et al. 2014; Bell et al. 2015), land surface models (Worden et al. 2025; Bilir et al. 2025; Zhang et al. 2022), and atmospheric transport models (Sorí et al. 2022; O'Connor et al. 2021).

**FIGURE 12:**
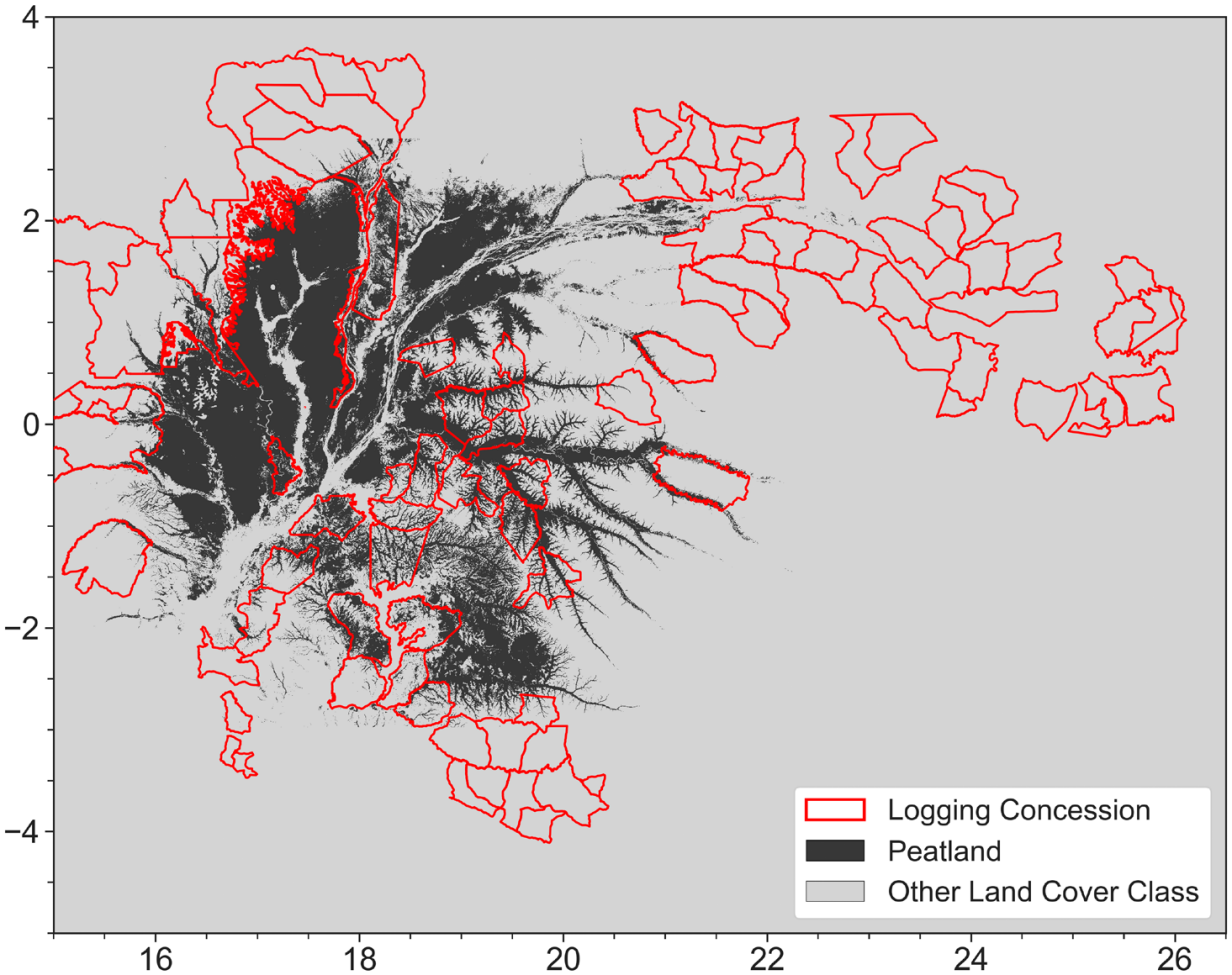
Map of Congo peatlands with logging concessions in red. Figure adapted from https://congopeat.net/maps/ (Dargie et al. 2019).

**FIGURE 13:**
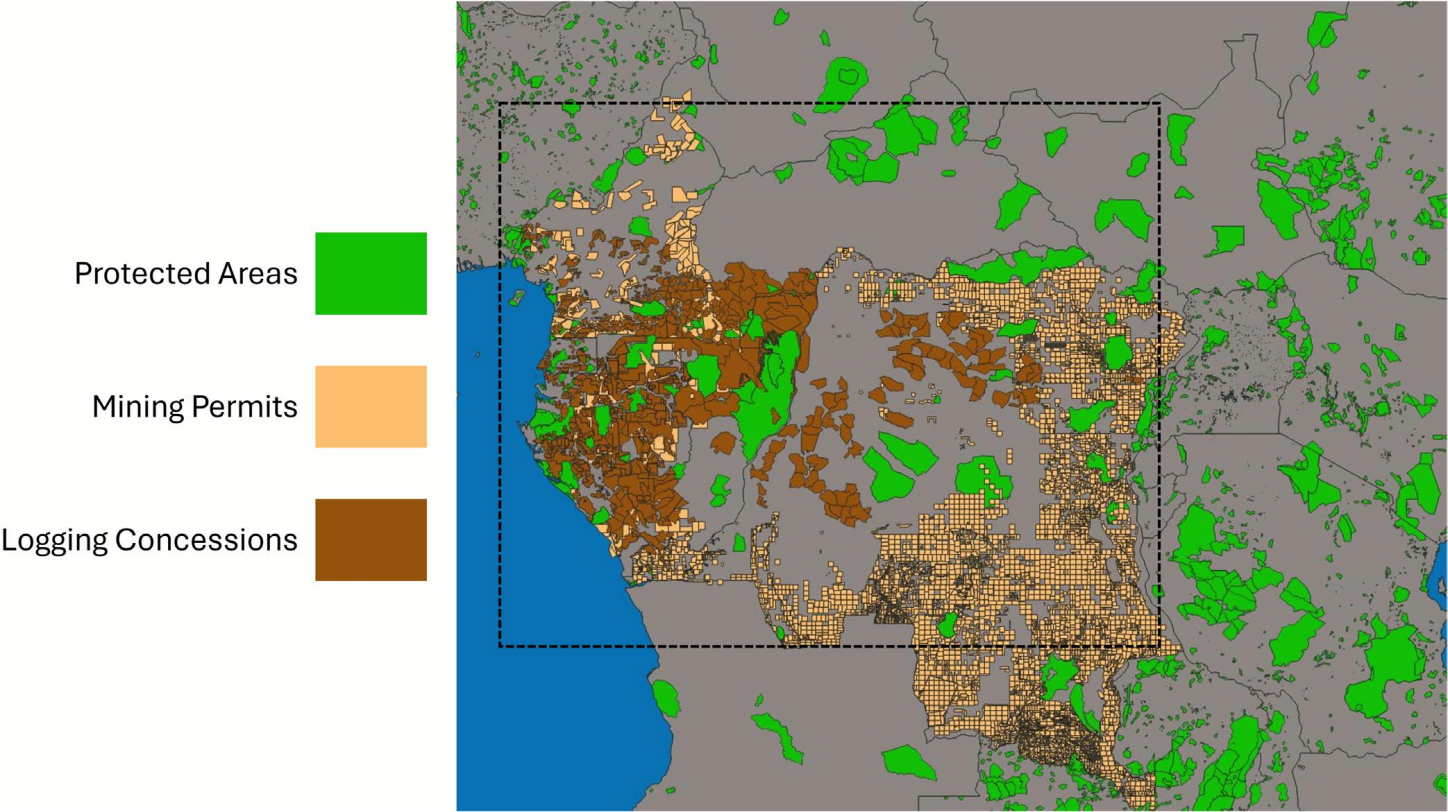
Protected areas (green), mining permits (tan), and logging concession areas (brown) in the equatorial African countries. All mining and logging data were downloaded from globalforestwatch.org. Protected areas were downloaded from http://protectedplantet.net (UNEP‐WCMC and IUCN 2025). Mining permit data were not available for Equatorial Guinea or the Central African Republic. Map lines delineate study areas and do not necessarily depict accepted national boundaries.

The scarcity of in situ weather station data (Tshimanga et al. 2021, 2022; Nicholson et al. 2018) limits understanding of vegetation response to rising temperatures. Projected end‐of‐century warming of 3°C–5°C (Crous et al. 2022; Malhi et al. 2013; Cook et al. 2025) may surpass the ~29°C–32°C optimum for net $CO_{2}$ assimilation in tropical forests (Réjou‐Méchain et al. 2021b; Wright 2010; Sullivan et al. 2020). A DRC study found a temperature optimum of 29.35°C ± 2.70°C, near the regional mean maximum of 29.8°C (Likoko et al. 2019; Lamotte 2024), suggesting some trees may already be near thermal limits. Additionally, temperature increases due to long‐term warming and LCLUC likely increase soil respiration due to increased microbial activity (Maes et al. 2024; Bond‐Lamberty et al. 2018; Davidson and Janssens 2006). This could have large impacts within the Cuvette Centrale peatland, of which over 26% is allocated to logging, mining, and palm oil concessions, and smallholder agricultural activities are large (> 88%) drivers of disturbances (Dargie et al. 2017, 2019; Nesha et al. 2024; Figures 12 and 13). Whether the combined effects of LCLUC and warming will reduce net carbon uptake across the Congo Basin's diverse ecosystems remains unclear.

Precipitation variability in the Congo Basin remains poorly understood, in part due to limited observations and weak model representation of rainfall processes (Washington et al. 2013; Castelvecchi 2025; Akinsanola et al. 2025). Precipitation is shaped by complex interactions between local and remote atmospheric circulations, and atmospheric moisture contributions from the forests via ET (Worden and Fu 2025; Nicholson 2022; Cook and Vizy 2022). Precipitation variability is linked to sea surface temperature variability in the tropical Atlantic, Pacific, and Indian Oceans (Balas et al. 2007; Farnsworth et al. 2011; Hua et al. 2016, 2018; Camberlin et al. 2001), driven by Indian Ocean Dipole variability (Moihamette et al. 2024), South Atlantic Ocean Dipole variability (Nana et al. 2024), and ENSO events (Camberlin et al. 2001). Because the Congo Basin overlaps with the West and South African Monsoons (Song et al. 2023; Lv et al. 2024; Wang and Ding 2008); precipitation variability should also be considered within the broader context of global monsoon changes (Zhisheng et al. 2015).

Observed precipitation changes include longer dry seasons (Jiang et al. 2019), larger, more frequent thunderstorms (Raghavendra et al. 2018; Alber et al. 2021), and long‐term declines in rainfall in April–June (Nicholson et al. 2022; Hua et al. 2016, 2018). Historical records (1937–1956) link deciduous phenology to seasonal rainfall (Kearsley et al. 2024), suggesting recent precipitation shifts may disrupt phenological cycles in a largely semi‐deciduous region. While Amazon‐based studies show storm–induced windthrows increase tree mortality, alter species composition, and reduce biomass (Feng et al. 2023; Urquiza‐Muñoz et al. 2024; Marra et al. 2018; Dos Santos et al. 2016), similar work is lacking in the Congo Basin where mesoscale convective systems provide most rainfall (Andrews et al. 2024). Furthermore, more frequent and intense storms (Taylor et al. 2018) could increase lateral carbon fluxes via sediment and nutrient exports within the Congo River (Baumgartner et al. 2022, 2023).

Deforestation‐induced changes to moisture recycling contribute to drying and, eventually, self‐reinforcing dieback in the Amazon (Staal et al. 2018, 2020; Zemp et al. 2017; Armstrong McKay et al. 2022; Xu et al. 2022; Flores et al. 2024; Qin et al. 2025). Similar factors may apply to the Congo Basin, where rainfall strongly depends on ET (Sorí et al. 2022; Worden, Fu, et al. 2021; Te Wierik et al. 2024; Staal et al. 2020; Smith, Baker, and Spracklen 2023; Van der Ent et al. 2010). Meanwhile, remote deforestation, degradation, and biomass burning to the north and south of the Congo Basin has important effects such as influencing cloud formation and lifetime via cloud‐aerosol interactions from biomass burning plumes transported over the region and nutrient input in the system that may modulate vegetation response to $CO_{2}$ fertilization (Mallet et al. 2024; Goll et al. 2023; Chaboureau et al. 2022). Understanding these interactions requires the utilization of ground‐based measurements, chronosequences (a space‐for‐time substitution), remote sensing, and modeling to investigate impacts of local and remote forest conversion on moisture recycling, surface temperatures, nutrient inputs, and more (Norden et al. 2015; Poorter et al. 2021; Rozendaal et al. 2019; Makelele 2022; Bauters, Vercleyen, et al. 2019).

## 9 Intersections With Sustainable Development

Despite Congo Basin forests representing one of the largest terrestrial above and belowground carbon reservoirs, their poorly quantified carbon cycle dynamics hampers informed conservation planning and management, both locally and internationally (Bele et al. 2015; Brown et al. 2010). Concurrently, the region is experiencing increased demand for resources from other nations, rapid development, and a growing population heavily dependent on land‐based sectors (Chamberlin et al. 2014; Byaro et al. 2023). Initiatives like the Yaoundé Declaration, Congo Basin Forest Partnership (CBFP), the Central African Forest Commission (COMIFAC) Convergence Plan, and the Central Africa World Heritage Forest Initiative (CAWHFI) for the Congo Basin have prioritized efforts to reduce deforestation, including the establishment of protected areas, agroforestry, and sustainable forest management (Kamdem‐Toham et al. 2003; Gauer 2006; Usongo and Nagahuedi 2007; Koyo 2004; Resende and Meikengang 2023; Brandt et al. 2016). Protected areas currently cover roughly 15% of African tropical forests (Resende and Meikengang 2023; Figure 13), although the large Cuvette Centrale peatland complex remains under‐protected, heightening carbon emission risks (Dargie et al. 2019; Loisel et al. 2021; Sonwa, Lewis, et al. 2022; Sonwa, Bambuta, et al. 2022). In addition, protected area enforcement is undermined by chronic underfunding and pressures from poaching, illegal mining and logging, unsustainable harvesting, and agricultural encroachment (Angu et al. 2011; Daskin and Pringle 2018; Kipute et al. 2023; White et al. 2021; Eba'a Atyi et al. 2019; Muteya et al. 2024; Abernethy et al. 2016, Figure 13). This reflects the immense challenge of reconciling forest ecosystem conservation with development (Mubalama et al. 2020).

Sustainable forest management policies, such as mandatory forest management plans (FMPs) in logging concessions, have reduced deforestation by rotating timber extraction cycles and closing old logging roads (Tritsch et al. 2020). However, weak legal frameworks, poor coordination, and limited enforcement lead to mixed outcomes, with some studies showing reduced deforestation but others showing no impact (Tegegne et al. 2016; Nkoulou et al. 2021; Bele et al. 2015; Tshimanga et al. 2021; Kipute et al. 2023; Chervier et al. 2024; Defourny et al. 2011; Brandt et al. 2016; Tritsch et al. 2020; Cerutti et al. 2011). Similarly, Forest Stewardship Council (FSC)‐certified concessions have not consistently lowered carbon emissions, although reduced‐impact logging practices may help (Umunay et al. 2019). Meanwhile, illegal artisanal logging is rising, sometimes surpassing legal rates. This has prompted countries to sign Voluntary Partnership Agreements (VPAs) to promote legal practices, although the capacity to implement these agreements varies (Piabuo et al. 2021).

In parallel, sustainable agriculture practices like agroforestry and conservation agriculture are expanding (Molua et al. 2014), including efforts to improve soil fertility and carbon sequestration with nitrogen‐fixing trees (Koutika et al. 2021; Kasongo et al. 2009; Tchichelle et al. 2017; Asaah et al. 2011; Bauters et al. 2015). Regional strategies such as the African Palm Oil Initiative (APOI; Ordway, Sonwa, et al. 2019) have been developed to reduce deforestation from commodity crops, but regular assessment of impact management plans is needed (Feintrenie 2014). However, smallholder agriculture tends to drive more deforestation in the region than large‐scale agro‐industrial producers (e.g., Ordway, Naylor, et al. 2019; Ordway, Sonwa, et al. 2019) and is linked to high carbon emissions due to inefficient production and milling systems. Yet smallholders often lack the resources needed to meet sustainability standards, limiting their participation in sustainable certification schemes (e.g., Roundtable on Sustainable Palm Oil, RSPO) (Acobta et al. 2023; Ayompe et al. 2021).

## 10 Conclusion

The Congo Basin remains a net carbon sink, yet its vulnerability and resiliency are changing due to four major drivers of change. Although lower than elsewhere in the tropics, deforestation and degradation, driven by small‐scale agriculture, logging, mining, and agro‐industrial expansion, are increasing, reducing soil and biomass carbon stocks and fluxes and altering species composition and lateral riverine carbon transport. Converting forests to cropland inflicts the greatest carbon losses and disrupts species composition and function, whereas low‐impact logging can cause smaller carbon declines. Biomass burning remains peripheral but is increasing along the Congo River and forest edges, linked to deforestation and higher temperatures and VPD.

In contrast, several factors may buffer Congo Basin forests against drought, warming, and deforestation‐ including drought adaptation, fire‐derived nutrients, plant–animal interactions, $CO_{2}$ fertilization, and promoting nature‐based approaches. Tree ring studies show iWUE increases driven by rising CO_2_, and models project enhanced carbon sequestration. Saharan dust and regional biomass burning supply N and P, making these nutrients potentially less limiting than in other tropical forests, though cation limitations persist.

All these factors interact in complex ways, producing net positive, negative, or neutral effects on the region's carbon cycle. Understanding these interactions is critical for effective forest restoration and conservation to ensure the region remains a sink, and to assess critical thresholds. Similar concerns exist in the Amazon, where intense deforestation, climate change–induced drying, and droughts have turned parts of the southern Amazon into a carbon source (Gatti et al. 2021). Quantifying the Congo Basin's carbon stocks and fluxes and accurately attributing their changes to drivers is imperative to understand the fate of the region's carbon sink, inform conservation efforts, and ultimately determine its impact on regional and global climate dynamics and local‐to‐global ecosystem services.

## Author Contributions

**Sarah Worden:** conceptualization, investigation, writing – original draft, methodology, validation, visualization, writing – review and editing, software, formal analysis, project administration, data curation, resources, funding acquisition, supervision. **Rong Fu:** conceptualization, funding acquisition, supervision, writing – original draft, writing – review and editing. **A. Anthony Bloom:** conceptualization, writing – original draft, writing – review and editing. **Marijn Bauters:** visualization, supervision, writing – original draft, writing – review and editing. **Hans Verbeeck:** supervision, writing – original draft, writing – review and editing. **Temilola Fatoyinbo:** writing – original draft, writing – review and editing. **Wannes Hubau:** writing – original draft, writing – review and editing. **Lydie‐Stella Koutika:** writing – original draft, writing – review and editing. **Steve Kwatcho Kengdo:** writing – original draft, writing – review and editing. **Sybryn L. Maes:** writing – original draft, writing – review and editing. **Vincent Medjibe:** writing – original draft, writing – review and editing. **Nicholas J. Russo:** visualization, writing – original draft, writing – review and editing. **Sassan Saatchi:** conceptualization, supervision, writing – original draft, writing – review and editing. **Le Bienfaiteur Sagang:** writing – original draft, writing – review and editing. **Thomas B. Smith:** conceptualization, writing – original draft, writing – review and editing. **Denis J. Sonwa:** writing – original draft, writing – review and editing. **Pascal Boeckx:** supervision, writing – original draft, writing – review and editing. **Elsa M. Ordway:** conceptualization, investigation, funding acquisition, writing – original draft, writing – review and editing, visualization, resources, supervision, project administration, formal analysis, methodology.

## Funding

This work was supported by the National Science Foundation (1917781), FWO (1203025N), NASA Postdoctoral Program Fellowship, and European Research Council.

## Conflicts of Interest

The authors declare no conflicts of interest.

## Supporting information


**Data S1:** gcb70688‐sup‐0001‐Supinfo.pdf.

## Data Availability

The data used in this study are the following: Vegetation map is from ESA World Cover 10 m 2021 v200 (Zanaga et al. 2022): https://doi.org/10.5281/zenodo.7254221. Floristic Types Map is from Réjou‐Méchain et al. (2021a): https://dataverse.cirad.fr/dataset.xhtml?persistentId=doi:10.18167/DVN1/UCNCA7. GPP data used to calculate the GPP budget are from Madani et al. (2020): https://daac.ornl.gov/cgi‐bin/dsviewer.pl?ds_id=1789. AGC data are from Xu et al. (2021): https://zenodo.org/records/4161694. SOC data are from the FAO and ITPS Global Soil Organic Carbon Map v1.5: https://data.apps.fao.org/catalog/dataset/7730e747‐eb73‐49c9‐bfe6‐84ebae718743. Peat Carbon Density is from Crezee et al. (2022): https://congopeat.net/maps/. 2 m temperature data from ERA5‐Land Hersbach et al. (2020) can be downloaded at https://cds.climate.copernicus.eu/. Precipitation data are from CHIRPS V2; Funk et al. (2015): https://www.chc.ucsb.edu/data/chirps. Potential Evapotranspiration comes from GLEAM; Martens et al. (2017): https://www.gleam.eu/. Annual forest change comes from the Tropical Forest Monitoring Project (*Source:* EC JRC; Vancutsem et al. 2021): https://forobs.jrc.ec.europa.eu/TMF/data. Burned area is from GFED5; Chen et al. (2023a): https://zenodo.org/records/7668424. Map of Congo peatlands with logging concessions and oil palm concessions is from Dargie et al. (2019): Taken from https://congopeat.net/maps/. Mining and Logging Data: Downloaded from globalforestwatch.org. Protected Area Data; UNEP‐WCMC and IUCN 2025: Downloaded from protectedplanet.net.
