# Supplementary material for: Congo Basin Carbon Cycle Responses to Global Change
Source: Glob Chang Biol. 2026 Jan 20;32(1):e70688. doi: 10.1111/gcb.70688 (PMC12817162; doi:10.1111/gcb.70688)
Supplement: Supplementary file 1 — Data S1: gcb70688‐sup‐0001‐Supinfo.pdf. [file GCB-32-e70688-s001.pdf]

Congo Basin Carbon Cycle Responses to Global Change
Supplementary

Contents of This File:

Table S1-S3
Figure S1

		Carbon Density $tC\ ha^{-1}$	Coordinates or Location	Reference
AGC	Primary	231.48	$1.43\ N,\ 28.59\ E$	Makana et al., 2011
		191 ± 28.3	$0.29\ N,\ 25.31\ E$	Doetterl et al., 2015
		162 ± 20	$0.88\ N,\ 24.516\ E$	Kearsley et al., 2013
		163 ± 18.8	$0.79\ N,\ 24.50\ E$	Doetterl et al., 2015
		183.00	$0.78\ N,\ 24.50\ E$	Van de Perre et al., 2018
		220.35	$1.15\ N,\ 21.61\ E$	Bauters et al., 2019
		176.02 ± 27.35	$4.50\ N,\ 18.55\ E$	Gourlet-Fluery et al., 2013
		173 ± 91.96	$2.53\ N,\ 17.33\ E$	Ekoungoulou et al., 2018
		132.05	$2.58\ N,\ 16.59\ E$	Day et al., 2014
		185.04	$2.81\ N,\ 16.15\ E$	Day et al., 2014
		121.75	$3.55\ N,\ 15.93\ E$	Gourlet-Fleury et al., 2011
		123.76 ± 34.32	$2.29\ N,\ 15.86\ E$	Zapfack et al., 2013
		137.57	$2.91\ N,\ 15.55\ E$	Neba et al., 2013
		168.60	$3.26\ S,\ 15.46\ E$	Ekoungoulou et al., 2015
		128.35	$3.27\ S,\ 15.45\ E$	Ekoungoulou et al., 2021
		170.67	$4\ S,\ 14\ E$	Ekoungoulou et al., 2014a
		180.99 ± 25.8	$4.52\ S,\ 13.78\ E$	Zekeng et al., 2020
		193.7 ± 21.6	$5.33\ N,\ 13.48\ E$	Mokake et al., 2023
		146.17	$0.79\ N,\ 13.30\ E$	Mankou et al., 2017
		$134-238$	$2.59\ N,\ 12.75\ E$	Fongnzossie et al., 2014
		178.6 ± 40.79	$0.25\ S,\ 12.75\ E$	Medjibe et al., 2013
		141.94	$1.76\ S,\ 12.27\ E$	Gautam and Pietsch 2012
		118	$4.50\ N,\ 11.17\ E$	Nijmeijer et al., 2018
		$122-282$	$3.31\ N,\ 10.63\ E$	Gonmadje et al., 2017
		214.19	$0.8\ N,\ 10.59\ E$	Day et al., 2014
		197.58	$0.33\ N,\ 10.33\ E$	Medjibe et al., 2011
		180.34	$1.50\ N,\ 10.25\ E$	Day et al., 2014
		117.65	$2.52\ N,\ 10.11\ E$	Day et al., 2014

		136 ± 36		$2.55\ S,\ 9.77\ E$	Kauffman and Bhomia 2017
		$2.5\ -\ 189.2$		$0.16\ N,\ 9.63\ E$	Trettin et al., 2021
		180 ± 40		$0.58\ N,\ 9.56\ E$	Kauffman and Bhomia 2017
		165.03		$6.17\ N,\ 9.34\ E$	Day et al., 2014
		115.93		$5.57\ N,\ 8.98\ E$	Day et al., 2014
	Secondary	85.52 ± 5.2		$0.28\ N,\ 25.30\ E$	Makelele et al., 2022
		106.3 ± 17.7		$1.15\ N,\ 21.61\ E$	Bauters et al., 2019
		118.60 ± 36.62		$2.29\ N,\ 15.86\ E$	Zapfack et al., 2013
		135.97		$4\ S,\ 14\ E$	Ekoungoulou et al., 2014b
		318.58 ± 54		$5.33\ N,\ 13.48\ E$	Kabelong Banoho et al., 2018
		$144\ -\ 245$		$2.59,\ 12.75\ E$	Fongnzossie et al., 2014
		125.2 ± 23.4		$3.98\ N,\ 12.47\ E$	Silatsa al., 2017
		107.61		$4.5\ S,\ 12.21\ E$	Nzala et al., 2019
BGC	Primary	40.65 ± 5.08		$2.53\ N,\ 17.33\ E$	Ekoungoulou et al., 2018
		37.17 ± 12.32		$2.29\ N,\ 15.86\ E$	Zapfack et al., 2013
		39.55		$3.26\ S,\ 15.46\ E$	Ekoungoulou et al., 2015
		64.16		$3.27\ S,\ 15.45\ E$	Ekoungoulou et al., 2020
		45.65		$4.52\ S,\ 13.78\ E$	Zekeng et al., 2020
		75.0 ± 8.8		$5.33\ N,\ 13.48\ E$	Mokake et al., 2023
		28.1 ± 10		$1.76\ S,\ 12.27\ E$	Gautam and Pietsch 2012
	Secondary	36.67 ± 21.02		$2.29\ N,\ 15.86\ E$	Zapfack et al., 2013
		31.95		$4\ S,\ 14\ E$	Ekoungoulou et al., 2014b
		74.16 ± 25.4		$5.33\ N,\ 13.48\ E$	Kabelong Banoho et al., 2018
		22.06		$4.5\ S,\ 12.21\ E$	Nzala et al., 2019
SOC	Primary	109.5 ± 21.4	0-90 cm	$0.79\ N,\ 24.50\ E$	Doetterl et al., 2015
		111 ± 24	0-100 cm	$0.88\ N,\ 24.51\ E$	Kearsley et al., 2013
		164.7 ± 15.9	0-100 cm	$1.15\ N,\ 21.61\ E$	Bauters et al., 2019
		116.16	0-15 cm	$1.79\ N,\ 17.97\ E$	Ifo et al., 2017
		17.21	0-15 cm	$1.79\ N,\ 17.97\ E$	Ifo et al., 2017
		43.92	0-15 cm	$1.79\ N,\ 17.97\ E$	Ifo et al., 2017
		155	0-100 cm	$2.63\ N,\ 15.99\ E$	Chiti et al., 2016

		39.39	0-20 cm	4.52 S, 13.78 E	Zekeng et al., 2020
		10.23±0.71	0-30 cm	5.33 N, 13.48 E	Mokake et al., 2023
		186.1±40.5	0-100 cm	1.76 S, 12.27 E	Gautam and Pietsch 2012
		51.50±2.62	10-30 cm	4.65 N, 11.67 E	Mandah et al., 2024
		135	0-100 cm	0.12 S, 11.60 E	Chiti et al., 2016
		58.9±19.6	0-60 cm	5.47 N, 10.65 E	Kenfack et al., 2024
		149.7±8.6	0-30 cm	5.60 N, 10.22 E	Wijungbwen et al., 2023
		274.2	0-100 cm	5.27 N, 9.93 E	Kome et al., 2021
		210.2±18.3	0-75 cm	5.67 N, 9.88 E	Tsozué et al., 2019
	Secondary	111.8±23.8	0-100 cm	1.15 N, 21.61 E	Bauters et al., 2019
		36.94	0-15 cm	1.79 N, 17.97 E	Ifo et al., 2017
		56.6±6.2	0-30 cm	3.98 N, 12.47 E	Silatsa et al., 2017
TBGC	Primary	392±103		2.55 S, 9.77 E	Kauffman and Bhomia 2017
		618.2 − 706.8		0.16 N, 9.63 E	Trettin et al., 2021
		866±46		0.58 N, 9.56 E	Kauffman and Bhomia 2017
		632.65		4.79 N, 8.73 E	Bumtu et al., 2020
ABGC	Primary	308		3.34 N, 11.65 E	Kotto-Same et al., 1997
	Secondary	250.12±55.48		4.96 N, 14.94 E	Temgoua et al., 2018

Table S1: Above and belowground carbon stocks reported by *in situ* studies with less than 10 reported coordinate locations. Secondary forest was reported if the study specifically noted "secondary" forest within. Coordinates represent the locations of the *in situ* studies, or the middle point of a reported range of coordinates. BGC is total belowground living biomass carbon. SOC is soil organic carbon, reported at different depths. Total BGC is total belowground carbon (living and dead) Some studies reported only total above and belowground carbon stocks, i.e., ABGC. A conversion factor of 0.47 was used if studies reported biomass rather than carbon stocks.

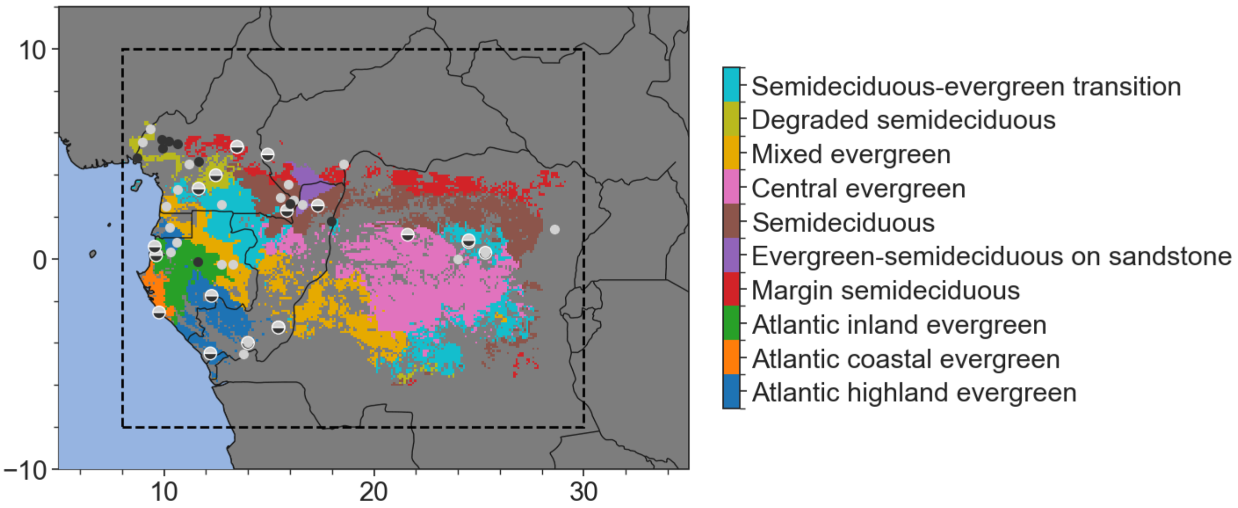

Figure S1: Locations of site-scale studies with less than 10 different coordinate locations of above and belowground carbon stocks in equatorial Africa (Table 1). Light grey circles represent aboveground carbon stocks, dark grey circles represent belowground carbon stocks, and grey/back circles indicate both were reported. The map is the floristic types from Réjou-Méchain et al., 2021.

		**Carbon Density** $tC\ ha^{-1}$	**Countries**	**Reference**
AGC	Primary	166.6	Gabon	Poulsen et al., 2020
		149.4 ± 13.55	Cameroon, DRC	Cuni-Sanchez et al., 2021
		252.86	Cameroon, Gabon, RoC, DRC	Ajonina et al., 2014
		$274 - 305$	Cameroon, Gabon, RoC, DRC	Lewis et al., 2013
		$125.60 - 242.50$	Cameroon	Nolte et al., 2001
		147.70 ± 69.70	RoC	Bocko et al., 2017
		95.30 ± 13.20	RoC	Dargie et al., 2019
	Secondary	96.6	Gabon	Poulsen et al., 2020
BGC	Primary	$33.0 - 57.40$	DRC	Xu et al., 2017
		89.77	Cameroon, Gabon, RoC, DRC	Ajonina et al., 2014
SOC	Primary	$90.0\ -$<br>0-200 cm	Central Africa	Batjes 2008

		253.0			
		129	0-200 cm	RoC	Schwartz and Namri 2002
		163	0-200 cm	Gabon	Wade et al., 2019
		170.37 ± 9.77	0-30 cm	Cameroon	Kum et al., 2021
		967.4 ± 57.6	0-100 cm	Cameroon, Gabon, RoC, DRC	Ajonina et al., 2014
	Secondary	56.6 ± 6.2	0-30 cm	Cameroon	Silatsa et al., 2020
Peat Carbon	Primary	2186		RoC	Dargie et al., 2017
		1712±634		RoC, DRC	Crezee et al., 2022

Table S2: Above and belowground carbon stocks reported by *in situ* studies with more than 10 reported coordinate locations. Secondary forest was reported if the study specifically noted "secondary" forest within.

	Carbon Flux $MtC\ yr$	River or Basin	Reference
DOC	0.4	Oubangui	Seyler et al., 2005
	0.54	Oubangui River	Coynel et al., 2005
	0.70	Oubangui River	Bouillon et al., 2012
	3.1	Kasai	Seyler et al., 2005
	1.2	Ngoko/Sangha+Likouala	Seyler et al., 2005
	0.26	Ngoko River	Coynel et al., 2005
	6.5	Upper Zaire	Seyler et al., 2005
	0.3	Bateke Rivers	Seyler et al., 2005
	11.5	Congo-Zaire	Seyler et al., 2005
	12.38	Congo-Zaire River	Coynel et al., 2005
	12.48	Congo River	Spencer et al., 2016
	16.2	Congo River	N’kaya et al., 2020
	15	Congo River	Laraque et al., 2013
	0.02	Mpoko River	Coynel et al., 2005
	2.48	Ruki River	Drake et al., 2023
DIC	3.73	Congo River	Wang et al., 2013
	0.49	Oubangui River	Bouillon et al., 2012
POC	0.2	Oubangui	Seyler et al., 2005
	0.15	Oubangui River	Coynel et al., 2005
	0.14	Oubangui River	Bouillon et al., 2012
	0.4	Kasai	Seyler et al., 2005
	0.3	Ngoko/Sangha+Likouala	Seyler et al., 2005
	0.06	Ngoko River	Coynel et al., 2005
	1	Upper Zaire	Seyler et al., 2005
	0.1	Bateke Rivers	Seyler et al., 2005
	1.99	Congo-Zaire River	Coynel et al., 2005

	1.9	Congo-Zaire	Seyler et al., 2005
	1.96	Congo River	Spencer et al., 2016
	0.01	Mpoko River	Coynel et al., 2005
CO_2 evasion	133-176	Congo River	Borges et al., 2015
	251±46	Congo River	Borges et al., 2019

Table S3: Dissolved Organic Carbon (DOC), Dissolved Inorganic Carbon (DIC) Particulate Organic Carbon (POC), and CO_2 evasion within the Congo Basin. Total carbon fluxes, rather than total carbon flux density is reported.

References

1. Ajonina, G. N., Kairo, J., Grimsditch, G., Sembres, T., Chuyong, G., & Diyouke, E. (2014). Assessment of mangrove carbon stocks in Cameroon, Gabon, the Republic of Congo (RoC) and the Democratic Republic of Congo (DRC) including their potential for reducing emissions from deforestation and forest degradation (REDD+). The land/ocean interactions in the coastal zone of West and Central Africa, 177-189. https://doi.org/10.1007/978-3-319-06388-1_15
2. Batjes, N. H. (2008). Mapping soil carbon stocks of Central Africa using SOTER. Geoderma, 146(1-2), 58-65. https://doi.org/10.1016/j.geoderma.2008.05.006
3. Bauters, M., Vercleyen, O., Vanlauwe, B., Six, J., Bonyoma, B., Badjoko, H., ... & Boeckx, P. (2019). Long‐term recovery of the functional community assembly and carbon pools in an African tropical forest succession. *Biotropica*, *51*(3), 319-329. https://doi.org/10.1111/btp.12647

4. Bocko, Y. E., Ifo, S. A., & Loumeto, J. J. (2017). Quantification des stocks de carbone de trois pools clés de carbone en Afrique centrale: Cas de la forêt marécageuse de la Likouala (Nord Congo). European Scientific Journal, 13(5), 438-456. https://doi.org/10.19044/esj.2017.v13n5p438k/
5. Borges, A. V., Darchambeau, F., Lambert, T., Morana, C., Allen, G. H., Tambwe, E., ... & Bouillon, S. (2019). Variations in dissolved greenhouse gases (CO 2, CH 4, N 2 O) in the Congo River network overwhelmingly driven by fluvial-wetland connectivity. Biogeosciences, 16(19), 3801-3834. https://doi.org/10.5194/bg-16-3801-2019
6. Borges, A. V., Darchambeau, F., Teodoru, C. R., Marwick, T. R., Tamooh, F., Geeraert, N., ... & Bouillon, S. (2015). Globally significant greenhouse-gas emissions from African inland waters. Nature Geoscience, 8(8), 637-642.https://doi.org/10.1038/ngeo2486
7. Bumtu, K. P., Nkwatoh, A. F., & Longonje, S. N. (2020). A Baseline Assessment of Soil Organic Carbon in the Mangroves of the Bakassi Peninsula South-West Cameroon. Published in Int J Trend Sci Res Dev (IJTSRD), 4(3), 414-421.
8. Chiti, T., Perugini, L., Vespertino, D., & Valentini, R. (2016). Effect of selective logging on soil organic carbon dynamics in tropical forests in central and western Africa. Plant and soil, 399, 283-294. https://doi.org/10.1007/s11104-015-2697-9
9. Coynel, A., Seyler, P., Etcheber, H., Meybeck, M., & Orange, D. (2005). Spatial and seasonal dynamics of total suspended sediment and organic carbon species in the Congo River. Global biogeochemical cycles, 19(4). https://doi.org/10.1029/2004GB002335
10. Crezee, B., Dargie, G. C., Ewango, C. E., Mitchard, E. T., Emba B, O., Kanyama T, J., ... & Lewis, S. L. (2022). Mapping peat thickness and carbon stocks of the central Congo

Basin using field data. Nature Geoscience, 15(8), 639-644. https://doi.org/10.1038/s41561-022-00966-7

11. Cuni-Sanchez, A., Sullivan, M. J., Platts, P. J., Lewis, S. L., Marchant, R., Imani, G., ... & Zibera, E. (2021). High aboveground carbon stock of African tropical montane forests. Nature, 596(7873), 536-542. https://doi.org/10.1038/s41586-021-03728-4
12. Dargie, G., Lewis, S., Lawson, I.., Mitchard, E.T.A., Page, S., Bocko, Y., et al. (2017) Age, extent and carbon storage of the central Congo Basin peatland complex. Nature 542, 86–90. https://doi.org/10.1038/nature21048
13. Day, M., Baldauf, C., Rutishauser, E., & Sunderland, T. C. (2014). Relationships between tree species diversity and above-ground biomass in Central African rainforests: implications for REDD. Environmental Conservation, 41(1), 64-72. doi:10.1017/S0376892913000295
14. Doetterl, S., Kearsley, E., Bauters, M., Hufkens, K., Lisingo, J., Baert, G., ... & Boeckx, P. (2015). Aboveground vs. belowground carbon stocks in African tropical lowland rainforest: Drivers and implications. *PloS one*, *10*(11), e0143209. https://doi.org/10.1371/journal.pone.0143209
15. Drake, T. W., Barthel, M., Mbongo, C. E., Mpambi, D. M., Baumgartner, S., Botefa, C. I., ... & Six, J. (2023). Hydrology drives export and composition of carbon in a pristine tropical river. Limnology and Oceanography, 68(11), 2476-2491. https://doi.org/10.1002/lno.12436
16. Ekoungoulou, R., Liu, X., Loumeto, J. J., Ifo, S. A., Bocko, Y. E., Koula, F. E., & Niu, S. (2014a). Tree allometry in tropical forest of Congo for carbon stocks estimation in above-ground biomass. Open Journal of Forestry, 4(05), 481. 10.4236/ojf.2014.45052

17. Ekoungoulou, R., Liu, X., Ifo, S. A., Loumeto, J. J., & Folega, F. (2014b). Carbon stock estimation in secondary forest and gallery forest of Congo using allometric equations. International Journal of Scientific and Technology Research, 3(3), 465-474.
18. Ekoungoulou, R., Mikouendanandi, M. R. B. E., & Liu, X. D. (2021). Carbon storage in an intact republic of Congo's forest. Applied Ecology and Environmental Research, 19(1), 439-451. DOI: http://dx.doi.org/10.15666/aeer/1901
19. Ekoungoulou, R., Niu, S., Loumeto, J. J., Ifo, S. A., Bocko, Y. E., Mikieleko, F. E. K., ... & Liu, X. (2015). Evaluating the carbon stock in above-and below-ground biomass in a moist central African forest. Applied Ecology and Environmental Sciences, 3(2), 51-59. doi: 10.12691/aees-3-2-4.
20. Ekoungoulou, R., Nzala, D., Liu, X., & Niu, S. (2018). Tree biomass estimation in central African forests using allometric models. Open Journal of Ecology, 8(3), 209-237. doi: 10.4236/oje.2018.83014.
21. Fongnzossie, E. F., Sonwa, D. J., Kemeuze, V., Auzel, P., & Nkongmeneck, B. A. (2014). Above‐ground carbon assessment in the K om‐M engamé forest conservation complex, South Cameroon: Exploring the potential of managing forests for biodiversity and carbon. In Natural Resources Forum (Vol. 38, No. 3, pp. 220-232). https://doi.org/10.1111/1477-8947.12049
22. Gautam, S., & Pietsch, S. A. (2012). Carbon pools of an intact forest in Gabon. African Journal of Ecology, 50(4), 414-427. https://doi.org/10.1111/j.1365-2028.2012.01337.x
23. Gonmadje, C., Picard, N., Gourlet-Fleury, S., Réjou-Méchain, M., Freycon, V., Sunderland, T., ... & Doumenge, C. (2017). Altitudinal filtering of large-tree species

explains above-ground biomass variation in an Atlantic Central African rain forest. Journal of Tropical Ecology, 33(2), 143-154. doi:10.1017/S0266467416000602

24. Gourlet-Fleury, S., Mortier, F., Fayolle, A., Baya, F., Ouédraogo, D., Bénédet, F., & Picard, N. (2013). Tropical forest recovery from logging: a 24 year silvicultural experiment from Central Africa. Philosophical Transactions of the Royal Society B: Biological Sciences, 368(1625), 20120302. https://doi.org/10.1098/rstb.2012.0302
25. Gourlet‐Fleury, S., Rossi, V., Rejou‐Mechain, M., Freycon, V., Fayolle, A., Saint‐André, L., ... & Picard, N. (2011). Environmental filtering of dense‐wooded species controls above‐ground biomass stored in African moist forests. *Journal of Ecology*, *99*(4), 981-990. https://doi.org/10.1111/j.1365-2745.2011.01829.x
26. Ifo, S. A., Koubouana, F., Binsangou, S., Parfait, A., & Marcelle, B. (2017). Amount of Soil Carbon Stock within Primary and Secondary Forest in the North of the Republic of Congo. Sustainability in Environment, 2(2), 159-170. http://dx.doi.org/10.22158/se.v2n2p159
27. Kabelong Banoho, L. P. R., Zapfack, L., Weladji, R. B., Chimi Djomo, C., Nyako, M. C., Bocko, Y. E., ... & Tabue Mbobda, R. B. (2020). Floristic diversity and carbon stocks in the periphery of Deng–Deng National Park, Eastern Cameroon. Journal of Forestry Research, 31, 989-1003. https://doi.org/10.1007/s11676-018-0839-7
28. Kauffman, J. B., & Bhomia, R. K. (2017). Ecosystem carbon stocks of mangroves across broad environmental gradients in West-Central Africa: Global and regional comparisons. PloS one, 12(11), e0187749. https://doi.org/10.1371/journal.pone.0187749
29. Kearsley, E., De Haulleville, T., Hufkens, K., Kidimbu, A., Toirambe, B., Baert, G., ... & Verbeeck, H. (2013). Conventional tree height–diameter relationships significantly

overestimate aboveground carbon stocks in the Central Congo Basin. *Nature communications*, *4*(1), 2269. https://doi.org/10.1038/ncomms3269

30. Kenfack, F. A. S., Kome, G. K., Ibrahim, A. B., Mandah, V. P., & Bitondo, D. (2024). Soil Organic Carbon Stock Variation under Different Soil Types and Land Uses in the Sub-Humid Noun Plain, Western Cameroon. Open Journal of Soil Science, 14(4), 191-209. DOI: 10.4236/ojss.2024.144011
31. Kome, G. K., Enang, R. K., & Yerima, B. P. K. (2021). Soil organic carbon distribution in a humid tropical plain of Cameroon: Interrelationships with soil properties. Applied and Environmental Soil Science, 2021(1), 6052513. https://doi.org/10.1155/2021/6052513
32. Kotto-Same, J., Woomer, P. L., Appolinaire, M., & Louis, Z. (1997). Carbon dynamics in slash-and-burn agriculture and land use alternatives of the humid forest zone in Cameroon. Agriculture, Ecosystems & Environment, 65(3), 245-256. https://doi.org/10.1016/S0167-8809(97)00060-1
33. Kum, C. T., Tening, A. S., Ngwabie, M., & Tsamo, C. (2021). Variation in Total Soil Organic Carbon Stocks in Relation to Some Land Use Systems in the Bamenda Highlands, Cameroon. Journal of Geoscience and Environment Protection, 9(9), 150-165. DOI: 10.4236/gep.2021.99009
34. Laraque, A., Castellanos, B., Steiger, J., Lòpez, J. L., Pandi, A., Rodriguez, M., ... & Lagane, C. (2013). A comparison of the suspended and dissolved matter dynamics of two large inter‐tropical rivers draining into the Atlantic Ocean: The Congo and the Orinoco. Hydrological Processes, 27(15), 2153-2170. https://doi.org/10.1002/hyp.9776
35. Lewis, S. L., Sonké, B., Sunderland, T., Begne, S. K., Lopez-Gonzalez, G., Van Der Heijden, G. M., ... & Zemagho, L. (2013). Above-ground biomass and structure of 260

African tropical forests. Philosophical Transactions of the Royal Society B: Biological Sciences, 368(1625), 20120295. https://doi.org/10.1098/rstb.2012.0295

36. Makana, J. R., Ewango, C. N., McMahon, S. M., Thomas, S. C., Hart, T. B., & Condit, R. (2011). Demography and biomass change in monodominant and mixed old-growth forest of the Congo. Journal of Tropical Ecology, 27(5), 447-461. doi:10.1017/S0266467411000265
37. Makelele, I. A., Bauters, M., Verheyen, K., Barthel, M., Six, J., Rütting, T., ... & Boeckx, P. (2022). Conservative N cycling despite high atmospheric deposition in early successional African tropical lowland forests. Plant and Soil, 477(1), 743-758.https://doi.org/10.1007/s11104-022-05473-7
38. Mandah, V. P., Tematio, P., Onana, A. A., Fiaboe, K. K., Arthur, E., Giweta, M. H., ... & Masso, C. (2024). Variability of soil organic carbon and nutrient content across land uses and agriculturally induced land use changes in the forest-savanna transition zone of Cameroon. Geoderma Regional, 37, e00808. https://doi.org/10.1016/j.geodrs.2024.e00808
39. Mankou, G. S., Picard, N., Ngomanda, A., & Loumeto, J. J. (2017). Co-variation in biomass and environment at the scale of a forest concession in central Africa. Journal of Tropical Ecology, 33(4), 249-260. doi:10.1017/S0266467417000177
40. Medjibe, V. P., Putz, F. E., & Romero, C. (2013). Certified and uncertified logging concessions compared in Gabon: changes in stand structure, tree species, and biomass. Environmental management, 51, 524-540. https://doi.org/10.1007/s00267-012-0006-4
41. Medjibe, V. P., Putz, F. E., Starkey, M. P., Ndouna, A. A., & Memiaghe, H. R. (2011). Impacts of selective logging on above-ground forest biomass in the Monts de Cristal in

Gabon. Forest Ecology and Management, 262(9), 1799-1806. https://doi.org/10.1016/j.foreco.2011.07.014

42. Mitchard, E. T., Saatchi, S. S., Lewis, S. L., Feldpausch, T. R., Woodhouse, I. H., Sonké, B., ... & Meir, P. (2011). Measuring biomass changes due to woody encroachment and deforestation/degradation in a forest–savanna boundary region of central Africa using multi-temporal L-band radar backscatter. Remote sensing of environment, 115(11), 2861-2873. https://doi.org/10.1016/j.rse.2010.02.022

43. Mitchard, E. T., Saatchi, S. S., White, L. J., Abernethy, K. A., Jeffery, K. J., Lewis, S. L., ... & Meir, P. (2012). Mapping tropical forest biomass with radar and spaceborne LiDAR in Lopé National Park, Gabon: overcoming problems of high biomass and persistent cloud. Biogeosciences, 9(1), 179-191.

44. Mokake, S. E., Weyi, B. K., Anyinkeng, N., Ngoh, L. M., Berkeley, O. E., & Andrew, E. E. (2023). Stand Diversity and Carbon Stock of a Tropical Forest in the Deng Deng National Park, Cameroon. Open Journal of Ecology, 13(7), 461-496. DOI: 10.4236/oje.2023.137029

45. Neba, S. G., Kanninen, M., Atyi, R. E. A., & Sonwa, D. J. (2014). Assessment and prediction of above-ground biomass in selectively logged forest concessions using field measurements and remote sensing data: Case study in South East Cameroon. Forest Ecology and Management, 329, 177-185.

46. Nijmeijer, A., Lauri, P. É., Harmand, J. M., & Saj, S. (2019). Carbon dynamics in cocoa agroforestry systems in Central Cameroon: afforestation of savannah as a sequestration opportunity. Agroforestry Systems, 93, 851-868. https://doi.org/10.1007/s10457-017-0182-6

47. Nolte, C., Kotto-Same, J., Moukam, A., Thenkabail, P. S., Weise, S. F., Woomer, P. L., & Zapfack, L. (2001). Land-use characterization and estimation of carbon stocks in the Alternatives to Slash-and-Burn benchmark area in Cameroon. Resource and Crop Management Research Monograph, 28, 25.

48. N'kaya, G. D. M., Orange, D., Bayonne Padou, S. M., Datok, P., & Laraque, A. (2020). Temporal variability of sediments, dissolved solids and dissolved organic matter fluxes in the Congo river at Brazzaville/Kinshasa. Geosciences, 10(9), 341. https://doi.org/10.3390/geosciences10090341

49. Nzala, D. (2019). Assessing the Floristic Biodiversity and Carbon Stock in a Republic of Congo's Forest Ecosystem. Open Access Library Journal, 6(08), 1. 10.4236/oalib.1105638

50. Poulsen, J. R., Medjibe, V. P., White, L. J., Miao, Z., Banak‐Ngok, L., Beirne, C., ... & Scott, C. T. (2020). Old growth Afrotropical forests critical for maintaining forest carbon. Global Ecology and Biogeography, 29(10), 1785-1798. https://doi.org/10.1111/geb.13150

51. Réjou-Méchain, M., Mortier, F., Bastin, J. F., Cornu, G., Barbier, N., Bayol, N., ... & Gourlet-Fleury, S. (2021). Unveiling African rainforest composition and vulnerability to global change. Nature, 593(7857), 90-94. https://doi.org/10.1038/s41586-021-03483-6

52. Schwartz, D., & Namri, M. (2002). Mapping the total organic carbon in the soils of the Congo. Global and Planetary change, 33(1-2), 77-93. https://doi.org/10.1016/S0921-8181(02)00063-2

53. Seyler, P., Coynel, A., Moreira-Turcq, P., Etcheber, H., Colas, C., Orange, D., ... & Meybeck, M. (2005). Organic carbon transported by the Equatorial rivers: example of

Congo-Zaire and Amazon basins. In Soil erosion and carbon dynamics (pp. 255-274). CRC Press.

54. Silatsa, F. B., Yemefack, M., Ewane-Nonga, N., Kemga, A., & Hanna, R. (2017). Modeling carbon stock dynamics under fallow and cocoa agroforest systems in the shifting agricultural landscape of Central Cameroon. Agroforestry systems, 91, 993-1006. https://doi.org/10.1007/s10457-016-9973-4
55. Silatsa, F. B., Yemefack, M., Tabi, F. O., Heuvelink, G. B., & Leenaars, J. G. (2020). Assessing countrywide soil organic carbon stock using hybrid machine learning modelling and legacy soil data in Cameroon. Geoderma, 367, 114260. https://doi.org/10.1016/j.geoderma.2020.114260
56. Trettin, C. C., Dai, Z., Tang, W., Lagomasino, D., Thomas, N., Lee, S. K., ... & Fatoyinbo, T. E. (2021). Mangrove carbon stocks in Pongara National Park, Gabon. Estuarine, Coastal and Shelf Science, 259, 107432. https://doi.org/10.1016/j.ecss.2021.107432
57. Temgoua, L. F., Momo Solefack, M. C., Nguimdo Voufo, V., Tagne Belibi, C., & Tanougong, A. (2018). Spatial and temporal dynamic of land-cover/land-use and carbon stocks in Eastern Cameroon: a case study of the teaching and research forest of the University of Dschang. Forest Science and Technology, 14(4), 181-191. https://doi.org/10.1080/21580103.2018.1520743
58. Tsozué, D., Nghonda, J. P., Tematio, P., & Basga, S. D. (2019). Changes in soil properties and soil organic carbon stocks along an elevation gradient at Mount Bambouto, Central Africa. Catena, 175, 251-262. https://doi.org/10.1016/j.catena.2018.12.028

59. Van de Perre, F., Willig, M. R., Presley, S. J., Bapeamoni Andemwana, F., Beeckman, H., Boeckx, P., ... & Verheyen, E. (2018). Reconciling biodiversity and carbon stock conservation in an Afrotropical forest landscape. *Science advances*, *4*(3), eaar6603. DOI: 10.1126/sciadv.aar6603

60. Wade, A. M., Richter, D. D., Medjibe, V. P., Bacon, A. R., Heine, P. R., White, L. J., & Poulsen, J. R. (2019). Estimates and determinants of stocks of deep soil carbon in Gabon, Central Africa. Geoderma, 341, 236-248. https://doi.org/10.1016/j.geoderma.2019.01.004

61. Wang, Z. A., Bienvenu, D. J., Mann, P. J., Hoering, K. A., Poulsen, J. R., Spencer, R. G., & Holmes, R. M. (2013). Inorganic carbon speciation and fluxes in the Congo River. Geophysical Research Letters, 40(3), 511-516. https://doi.org/10.1002/grl.50160

62. Wijungbwen, A. L., Nguemezi, C., Djeuhala, D. I. V., & Tematio, P. (2023). Soil Organic Carbon Stock and Soil Quality under Four Major Agroecosystems in the Eastern Flank of Mount Bambouto (West-Cameroon). Journal of Geoscience and Environment Protection, 11(9), 40-53. DOI: 10.4236/gep.2023.119004

63. Xu, L., Saatchi, S. S., Shapiro, A., Meyer, V., Ferraz, A., Yang, Y., ... & Ebuta, D. (2017). Spatial distribution of carbon stored in forests of the Democratic Republic of Congo. Scientific Reports, 7(1), 15030. https://doi.org/10.1038/s41598-017-15050-z

64. Xu, L., Saatchi, S., Yang, Y., Yu, Y., Pongratz, J., Bloom, A., et al. 2021. Changes in global terrestrial live biomass over the 21st century. Science Advances, 7(27): eabe9829. 18 p. https://doi.org/10.1126/sciadv.abe9829.

65. Zapfack, L., Noumi, V. N., Kwouossu, D. P., Zemagho, L., & Nembot, F. T. (2013). Deforestation and carbon stocks in the surroundings of Lobéké National Park (Cameroon) in the Congo Basin. Environment and Natural Resources Research, 3(2), 78.

66. Zekeng, J. C., van der Sande, M. T., Fobane, J. L., Mphinyane, W. N., Sebego, R., & Mbolo, M. M. A. (2020). Partitioning main carbon pools in a semi-deciduous rainforest in eastern Cameroon. Forest Ecology and Management, 457, 117686. https://doi.org/10.1016/j.foreco.2019.117686
